# ﻿Revision of the new Australasian orb-weaving spider genus *Salsa* (Araneae, Araneidae)

**DOI:** 10.3897/zookeys.1102.82388

**Published:** 2022-05-20

**Authors:** Volker W. Framenau, Pedro de S. Castanheira

**Affiliations:** 1 Harry Butler Institute, Murdoch University, 90 South St, Murdoch, Western Australia 6150, Australia Murdoch University Murdoch Australia; 2 Department of Terrestrial Zoology, Western Australian Museum, Locked Bag 49, Welshpool DC, Western Australia, 6986, Australia Western Australian Museum Welshpool Australia; 3 Zoological Museum Hamburg, Leibnitz Institute for the Analysis of Biodiversity Change (LIB), Centre for Taxonomy & Morphology, Martin-Luther-King-Platz 3, 20146 Hamburg, Germany Zoological Museum Hamburg Hamburg Germany

**Keywords:** Australia, backobourkiines, Pacific, South-east Asia, systematics, taxonomy

## Abstract

A new Australasian genus in the orb-weaving spider family Araneidae Clerck, 1757 is described to include seven species: *Salsafuliginata* (L. Koch, 1871) **comb. nov.** (type species; = *Epeirarubicundula* Keyserling, 1887) **syn. nov.**) (Australia, introduced to New Zealand); *S.brisbanae* (L. Koch, 1867) **comb. nov.** (Australia); *S.canalae* (Berland, 1924) **comb. nov.** (New Caledonia); *S.neneba***sp. nov.** (Papua New Guinea); *S.recherchensis* (Main, 1954) **comb. nov.** (Australia); *S.rueda***sp. nov.** (Australia); and *S.tartara***sp. nov.** (Australia; Lord Howe Island endemic). *Salsa***gen. nov.** belongs to the Australasian informal backobourkiine clade and differs from other genera of this clade by a distinct abdominal shape (single posterior abdominal tubercle) and ventral colouration (pale lateral spindle-shaped bands), male pedipalp morphology (C-shaped median apophysis that has teeth-like tubercles inside the basal arch) and the shape of the female epigyne scape (partially translucent and generally shorter than the epigyne plate). Based mainly on male pedipalp morphology within the backobourkiines, *Salsa***gen. nov.** has closest morphological affinities with *Acroaspis* Karsch, 1878 and *Socca* Framenau, Castanheira & Vink, 2022.

## ﻿Introduction

**Figure 1.**
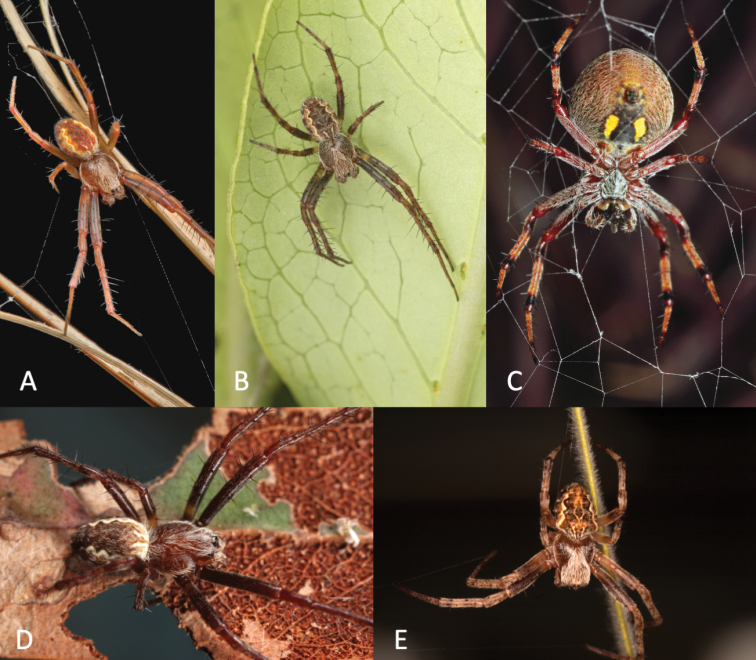
Life images of *Salsa* gen. nov. species **A–D***S.fuliginata* (L. Koch, 1872), comb. nov. **A** male, Brymer Park, Hamilton, New Zealand, North Island **B** male, Flagstaff, Hamilton, North Island, New Zealand **C** female, Rotorua North, Hamilton, North Island, New Zealand **D** male, Ringwood East, Victoria, Australia (WAM T100137) **E***S.recherchensis* (Main, 1954), comb. nov., female, Stirling Range National Park, Western Australia (WAM T81440). Images: **A–C** Bryce McQuillan **D, E** V.W. Framenau.

When Dondale (1966) transferred an Australian orb-weaving spider species from *Araneus* Clerck, 1757 to *Cyclosa* Menge, 1866, *C.fuliginata* (L. Koch, 1872) (e.g., Fig. 1A–D), he realised that this placement was not without problems as the carapace shape of males and females was unlike that of other *Cyclosa*. The problem was compounded by the fact that the first detailed diagnosis of the genus was not published until much later (Levi 1977). Chrysanthus (1961) had earlier reviewed some *Cyclosa* from south-east Asia but did not provide a diagnosis for the genus. With Levi’s (1977, 1999) reviews of the genus it became clear that the Australian species was misplaced in *Cyclosa* based on both somatic and genitalic characters, but no further taxonomic treatment of the species has been conducted since Dondale (1966).

Scharff et al.’s (2020) multi-loci molecular phylogenetic study of world-wide Araneidae Clerck, 1757 included many Australian species, but not *C.fuliginata* to facilitate its appropriate generic placement. A morphologically similar species, *Araneusrecherchensis* (Main, 1954) (e.g., Fig. 1E) represented a putative new genus (termed ‘NGEN02’) in that study, nesting with high support in a clade referred to as ‘backobourkiines’ and with closest affinities to *Acroaspis* Karsch, 1878, *Plebs* Joseph & Framenau, 2012 and *Socca* Framenau, Castanheira & Vink, 2022 (‘NGEN05’ in that study) (Scharff et al. 2020).

The Australian backobourkiines are currently the focus of a comprehensive taxonomic and systematic investigation with the aim to revise their constituent genera, but also to potentially characterise and diagnose this group as formal subfamily of the Araneidae based on both morphological and molecular data. This project has already identified a number of new genera, some of which were suggested by the molecular study of Scharff et al. (2020), such as *Hortophora* Framenau & Castanheira, 2021, *Socca* and others (e.g., Joseph and Framenau 2012; Framenau et al. 2021a, c, 2022).

Our morphological studies confirmed that *C.fuliginata* and *A.recherchensis* are indeed congeneric and belong to a new genus. The aim of this study is therefore to taxonomically revise this new genus of Australasian orb-weaving spiders as hypotheses for future systematic work on the backobourkiines.

## ﻿Materials and methods

Descriptions and terminology follow recent publications on Australian and New Zealand orb-weaving spiders (e.g., Joseph and Framenau 2012; Framenau et al. 2021a, b, c, 2022). Redescriptions of historically named species are based on recently collected, well-preserved material instead of the usually damaged and discoloured type specimens. Colour patterns were described based on specimens preserved in ca. 75% ethanol.

The description of the views of the male pedipalp relate to their position as a limb. A full view of the bulb with the cymbium in the background is a retrolateral view as in Araneidae the pedipalp is twisted so that the cymbium is situated mesally. Our standard views of the pedipalp therefore generally show the ventral view, to illustrate the diagnostic median apophysis, or the dorsal view with the tegulum in full view, as the cymbium is situated to the side in our images. Like in our recent papers (Framenau et al. 2021a, c, 2022), the term ‘conductor lobe’ is preferred over ‘paramedian apophysis’ for a structure originating at the base of the conductor in the male pedipalp (see also Framenau et al. 2010, 2021c for discussions on this sclerite). The designation of an apical structure of the pedipalp bulb as terminal apophysis is this study is consistent with Dondale’s (1966) application of this term and also Framenau et al.’s (2022) use for a similar, but tri-partite apophysis in *Socca*. In *Salsa* gen. nov., the terminal apophysis may carry two appendices, a basal ‘prong’ and an apical ‘process’. Our nomenclature, however, does not necessarily suggest homologies of these structures to those in other araneids but serves primarily to facilitate the description of the pedipalp morphology of males. Evaluating homologies of male pedipalp sclerites within the backobourkiines and against world-wide Araneidae will be the subject of future phylogenetic studies once all putative genera of backobourkiines have been revised. In this study, pedipalps were expanded by alternatively submerging them for 10 min in 10% KOH and distilled water until fully expanded.

The female epigyne consists of two main parts, the base (encapsulating the internal genitalia) and the scape. We refer to the central part of the base in ventral view as atrium which, in posterior view, becomes the central division. We cleared selected epigynes by submerging them in warm, 10% KOH for ca. 20 min. For observation and imaging, samples were transferred into lactic acid on a microscopic glass slide under a cover slip, which further cleared internal structures.

Throughout the course of this study, which commenced in 2005, microscopic photographs were taken with two different stereo-imaging systems. A setup at the Natural History Museum, Copenhagen (Denmark) allowed taking images with a Nikon D300 digital SLR camera attached via a C-mount adapter to a Leica M16A stereomicroscope. Images of different focal plains were stacked with Automontage (v. 5.02) software from Syncroscopy to increase depth of field. Two Nikon R1C1 wireless speedlights were used to illuminate the exposures. A second set-up at the Harry Butler Institute, Murdoch University (Australia) supported taking microscopic images in different focal planes (ca. 20–30 images) with a Leica DMC4500 digital camera mounted to a Leica M205C stereomicroscope and combined using the Leica Application Suite X, v. 3.6.0.20104. All photos were edited and mounted with Photoshop CC 2020.

All measurements are given in millimetres. They were taken with an accuracy of one tenth of a millimetre, with the exception of eye and labium measurements taken with an accuracy of one hundredth of a millimetre.

Maps were compiled in the software package QGis v. 2.14.0 Girona (https://qgis.org/en/site/; accessed 21 December 2021). Geographic coordinates were extracted directly from original labels or the registration data as provided by the museums. When no detailed geographic information was available, localities were estimated based on Google Earth v. 9.1.39.3 (https://earth.google.com/web/ accessed 21 December 2021) to the closest minute of Latitude and Longitude.

The taxonomic part of this study lists all species in alphabetical order, except for the type-species of the new genus, which is treated first.

### ﻿Abbreviations

**Morphology**:

**ALE** anterior lateral eyes;

**AME** anterior median eyes;

**PLE** posterior lateral eyes;

**PME** posterior median eyes;

**Collections**:

**AM**Australian Museum, Sydney (Australia);

**BNHM** Naturhistorisches Museum Basel (Switzerland);

**CMNZ**Canterbury Museum, Christchurch (New Zealand);

**CVIC**La Trobe University, Bendigo (Australia);

**LUNZ**Entomology Research Museum, Lincoln University (New Zealand);

**MONZ**Museum of New Zealand Te Papa Tongarewa, Wellington (New Zealand);

**MPI** Ministry of Primary Industries Manatū Ahu Matua, Auckland (New Zealand);

**MV** Museums Victoria, Melbourne, Australia;

**NHMD**Natural History Museum of Denmark, Zoological Museum, University of Copenhagen (Denmark);

**NHMUK**Natural History Museum, London (England, United Kingdom);

**NHMW**Naturhistorisches Museum, Wien (Austria);

**QM**Queensland Museum, Brisbane (Australia);

**QVMAG**Queen Victoria Museum & Art Gallery, Launceston (Australia);

**SAM** South Australian Museum, Adelaide (Australia);

**WAM**Western Australian Museum, Perth (Australia);

**ZMB**Museum für Naturkunde, Zentralinstitut der Humboldt-Universität, Berlin (Germany);

**ZMH**Zoologisches Institut und Zoologisches Museum, Universität Hamburg (Germany).

## ﻿Results

*Salsa* gen. nov. includes comparatively common species; a total of 263 males, 1,069 females (11 with egg sacs), and 321 juveniles in 616 records (i.e., vials) were examined for this study in Australian and overseas institutions (Table 1). *Salsa* gen. nov. contains seven species, five from Australia (of which one also occurs in New Zealand), one from New Caledonia, and one from Papua New Guinea (Table 1). In Australia, the highest diversity of *Salsa* gen. nov. is in the eastern states, where four of the five species occur. A single species, *S.recherchensis* comb. nov., is know from Western Australia and occurs into South Australia (Table 1).

**Table 1.** Summary of distribution, type specimen and other material examined and of species of *Salsa* gen. nov.

Species	Comments	Distribution	Type specimen	Other material examined
*S.fuliginata* (L. Koch, 1872), comb. nov.	Type species of *Salsa*; senior syn. of *E.rubicundula* (Keyserling)	NSW, SA, Tas, Vic; also New Zealand	Holotype female, Sydney (NSW) (NHMW)	162 males, 509 females (8 with egg sac), 105 juveniles (in 360 records)
*S.brisbanae* (L. Koch, 1867), comb. nov.		NSW, Qld, SA, Tas, Vic	Holotype female, Brisbane (Qld) (ZMHRack (1961)-catalogue no. 226)	57 males, 208 females (2 with egg sac), 39 juveniles (in 146 records)
*S.canalae* (Berland, 1924), comb. nov.		New Caledonia	Holotype female, Mount Canala (New Caledonia) (BNHM)	1 male, 7 females (in 8 records)
*S.neneba* sp. nov.		Papua New Guinea	Holotype female, Neneba (Papua New Guinea) (QM S111920)	
*S.recherchensis* (Main, 1954), comb. nov.		SA, WA	Holotype female, Fig. of Eight Island, Recherche Archipelago, (WA) (WAM 55/4984)	34 males, 321 females, 175 juveniles (in 74 records)
*S.rueda* sp. nov.		NSW, Tas, Vic	Holotype male, Tubrabucca (NSW) (MV K-14856)	6 males, 14 females (1 with egg sac), 1 juvenile (in 15 records)
*S.tartara* sp. nov.		NSW (endemic to Lord Howe Island)	Male holotype, Goat House Cave area, Lord Howe Island (NSW) (AM KS.70737)	1 male, 5 females (in 6 records)

Abbreviations: NSW – New South Wales, SA – South Australia, Tas – Tasmania, Vic – Victoria, WA – Western Australia.

### ﻿Taxonomy

#### Family Araneidae Clerck, 1757

##### ﻿ Salsa gen. nov.

Taxon classificationAnimaliaAraneaeAraneidae

6A46B084-7639-5BC6-A385-B1BE1A64240E

http://zoobank.org/92B3923D-E576-4925-B79C-85FD0F6CDBBB

###### Type species.

*Epeirafuliginata* L. Koch, 1872. Designated here.

###### Etymology.

The genus-group name refers to the Latin dance style Salsa, associated with the music genre of the same name. It is the favourite dance style of the senior author, but also a very popular dance style in Latin America, from where the junior author is. The name also refers to the Spanish/Italian word “salsa”, which means “sauce” or “gravy”. The gender of the genus-group name is feminine.

###### Diagnosis.

*Salsa* gen. nov. can only be properly diagnosed against the six backobourkiine genera that have been formally revised using modern taxonomic methods: *Backobourkia* Framenau, Dupérré, Blackledge & Vink, 2010, *Hortophora*, *Lariniophora* Framenau, 2011, *Novakiella* Court & Forster, 1993, *Plebs* and more recently *Socca* (Framenau et al. 2010; Framenau 2011; Joseph and Framenau 2012; Framenau et al. 2021a, c, 2022). Other established backobourkiine genera such as *Acroaspis*, *Carepalxis* L. Koch, 1872, and possibly *Singa* C.L. Koch, 1836 (see Scharff et al. 2020) are still awaiting revisions in Australia and without a modern circumscription of these genera, *Salsa* gen. nov. cannot be diagnosed from these.

**Figure 2.**
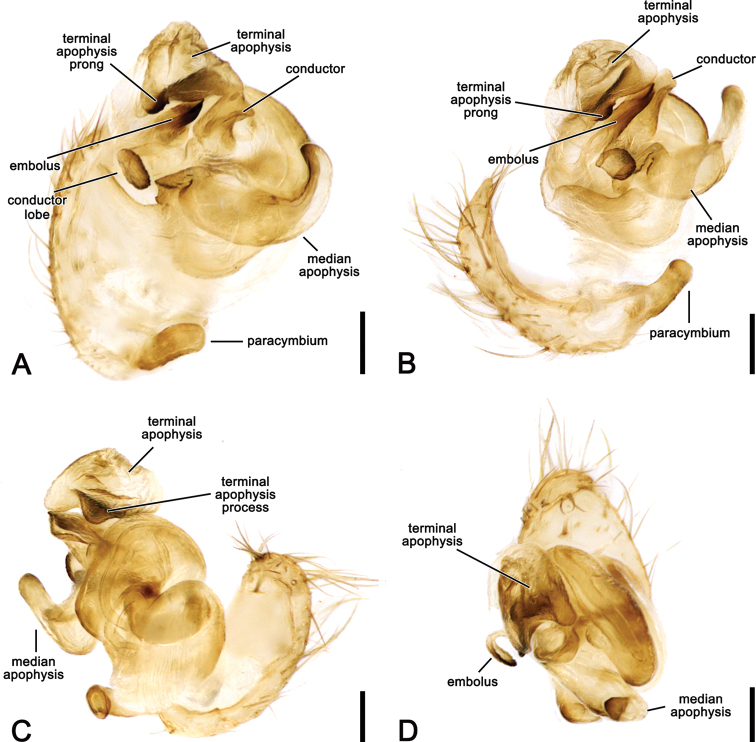
*Salsafuliginata* (L. Koch, 1872), comb. nov., expanded left pedipalp of male (MV K-14867 (CVIC 1163)) **A** ventral view **B** ventral view **C** dorsal view **D** apical view. Scale bars: 0.2 mm. Arrow in **A, B** points to the tubercle on median apophysis arch.

**Figure 3.**
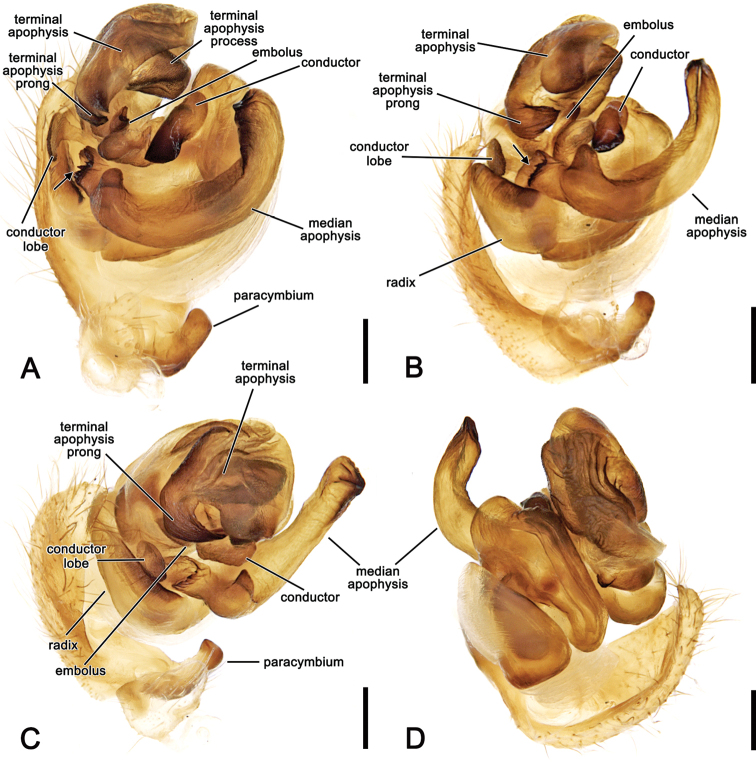
*Salsarueda* sp. nov., expanded left pedipalp of male holotype (MV K-14856) **A** ventral view **B** baso-ventral view **C** apico-ventral view **D** apical view. Arrow in **A, B** points to the tubercle on median apophysis arch. Scale bars: 0.2 mm.

**Figure 4.**
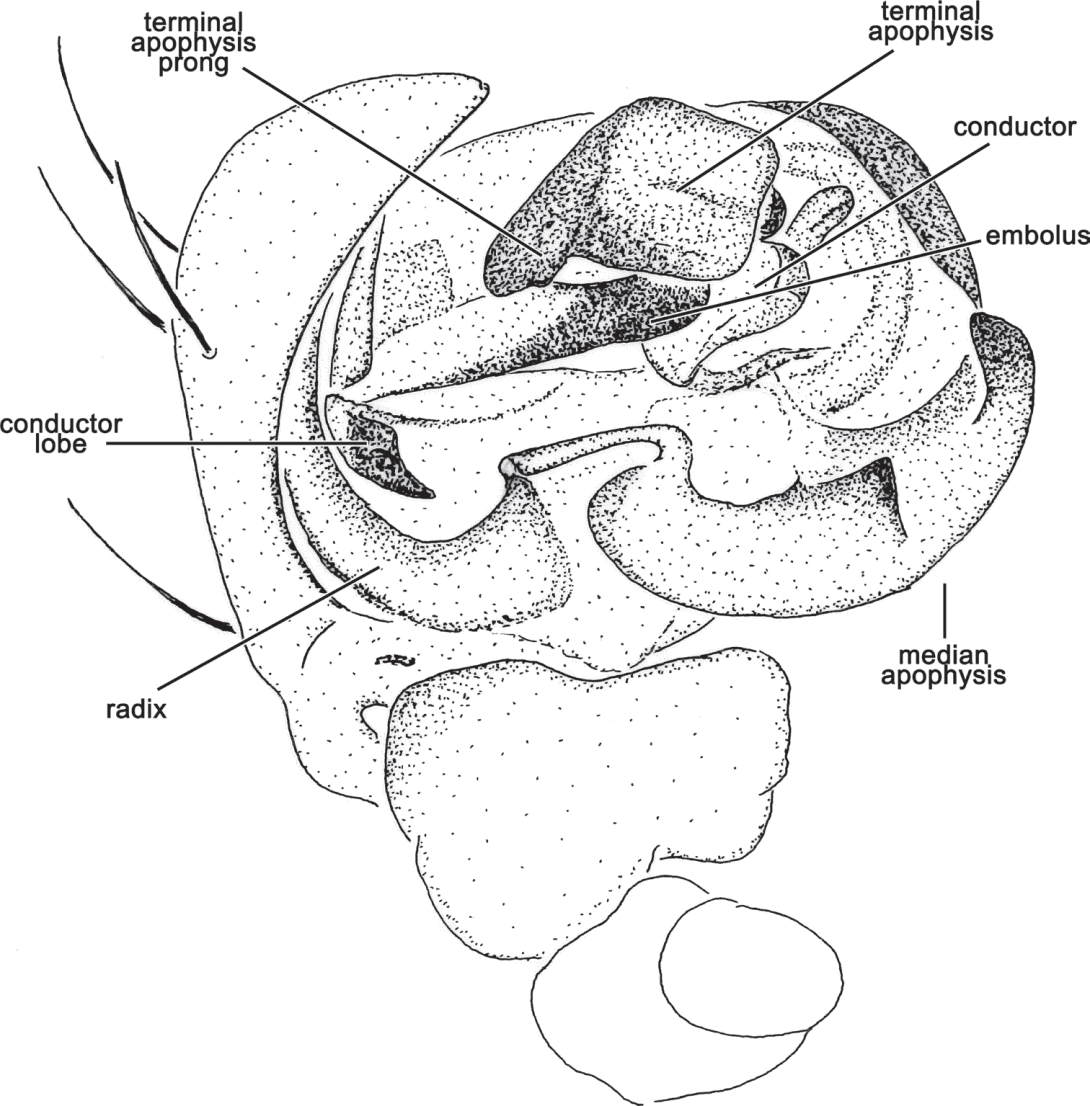
*Salsafuliginata* (L. Koch, 1872), comb. nov., male pedipalp, ventral view (WAM T67910). Scale bar: 0.2 mm.

We here identify the following synapomorphies to diagnose species in *Salsa* gen. nov. within the backobourkiines: single posterior abdominal tubercle (e.g., Figs 12A, 18A); venter with lateral pale elongate, ovoid, or spindle-shaped bands (e.g., Figs 6B, 7B, 9B, 10B); male pedipalp with C-shaped median apophysis and teeth-like tubercles inside its basal arch (e.g., Figs 2B, 3A–D, 4, 6C); female epigyne scape transparent and generally shorter than the epigyne plate (e.g., Figs 7C, D, 10C, 13C–E).

*Salsa* gen. nov. species differ from those of *Backobourkia* by the lack of a distinctive anterior triangular or comma-shaped white marking and the lack of strong spine-like setae found on the dorsum of the abdomen. Males of *Salsa* gen. nov. can be identified from those of *Backobourkia* by the absence of a basal flange on the median apophysis of the male pedipalp and females by the generally much wider atrium and central division on the epigyne (Framenau et al. 2010).

*Salsa* gen. nov. species differ from those of *Hortophora* in the generally smaller size (although sizes can sometimes overlap in smaller specimens of *Hortophora*); the shape of the median apophysis (C-shaped in *Salsa* gen. nov. but elongate transverse in *Hortophora* and generally with two apical tips), and the comparatively much shorter scape of the female epigyne (Framenau et al. 2021a).

The subtriangular to ovoid abdomen of *Salsa* gen. nov. greatly differs from the elongate abdomen of *Lariniophora*. *Salsa* gen. nov. males lack the bilobed outgrowth on the median apophysis characteristic for *Lariniophora*, and females lack the elevated epigyne base (Framenau 2011).

Male *Salsa* gen. nov. differ from those of *Novakiella* by the more elongate and curved median apophysis of the male pedipalp (shorter and pointing basally in *Novakiella*) and an inconspicuous conductor lobe (prominent in *Novakiella*) (Framenau et al. 2021c). The epigyne base in female *Novakiella* is triangular (Framenau et al. 2021c), whereas it is subquadrate in *Salsa* gen. nov.

Species of *Salsa* gen. nov. differ from those of *Plebs* by the less elongate abdomen and its ventral colouration, that has lateral bands in *Salsa* gen. nov. but an inverted Ü-shaped pattern in *Plebs* (Joseph and Framenau 2012). The median apophysis of male *Plebs* is elongate transverse with two apical tips (C-shaped with a single tip in *Salsa* gen. nov. males) and the female epigyne has a wider atrium and the scape is comparatively shorter in *Salsa* gen. nov. than it is in *Plebs*.

Species of *Salsa* gen. nov. differ from those of *Socca* by the number of posterior abdominal humps (one in *Salsa* gen. nov. and usually five in *Socca*), the shape of the terminal apophysis (distinctly tri-partite with central lamellar appendix in *Socca* but entire in *Salsa* gen. nov. sometimes with prong and process) (Framenau et al. 2022).

###### Description.

Median-sized orb-weaving spiders, males (ca. total length 3.2–6.1) smaller than females (ca. total length 6.5–10.5). Carapace longer than wide, pear-shaped and with cephalic region considerably narrower in males than in females; colouration variable from yellowish brown to reddish brown, normally covered with yellowish white setae (e.g., Figs 6A, 7A, 9A, 10A, 12A). Fovea longitudinal in males and transversal in females (e.g., Figs 6A, 7A, 9A, 10A, 12A). Anterior median eyes largest, row of posterior eyes slightly recurved, lateral eyes almost touching, posterior lateral eyes apart from posterior median eyes by more than their diameter; anterior median eyes slightly protruding from the carapace (e.g., Figs 6A, 7A, 9A, 10A, 12A). Sternum longer than wide (except on females of *S.canalae* comb. nov., in which it is as long as wide), comparatively narrower in males than females, with a sparse to dense cover of setae (e.g., Figs 6B, 7B, 9B, 10B, 12B). Labium wider than long, with anterior glabrous pale edge. Endites with glabrous paler antero-mesal section, that of males with lateral tooth. Chelicerae fangs with four promarginal teeth, of which the second-basal and/or apical are generally largest (reduced to three in *S.brisbanae* comb. nov. male and *S.fuliginata* comb. nov. male and female, with median largest), three retromarginal teeth with basal often largest. Legs (e.g., Figs 6A, B, 7A, B 9A, B): Leg formula I > II > IV > III. Abdomen slightly longer than wide, varying in shape from oval to sub-triangular, normally with inconspicuous humeral humps, abdomen otherwise without specialised setae, sigillae, condyles or other specific structures; colour dorsally with pale brown to beige background with variable darker folium pattern (Fig. 1A, B). Venter of variable colour, centrally generally darkest and generally with pale lateral ovoid, elongate or spindle-shaped bands (e.g., Figs 6A, B, 7A, B, 9A, B).

**Figure 5.**
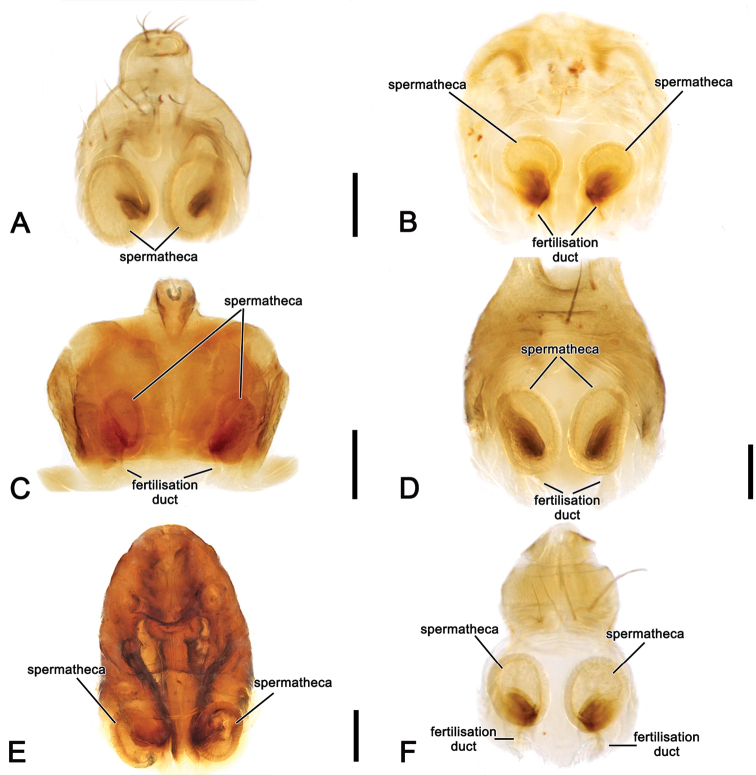
*Salsa* gen. nov. female epigynes, cleared posterior view **A***S.fuliginata* (L. Koch, 1872), comb. nov. (CVIC 173) **B***S.brisbanae* (L. Koch, 1867), comb. nov. (AM KS.131087) **C***S.canalae* comb. nov. Berland, 1924, comb. nov. (WAM T75921) **D***S.recherchensis* (Main, 1954), comb. nov. (WAM T77362) **E***S.rueda* sp. nov. **F***S.tartara* sp. nov. (AM KS.7061). Scale bars: 0.2 mm.

**Figure 6.**
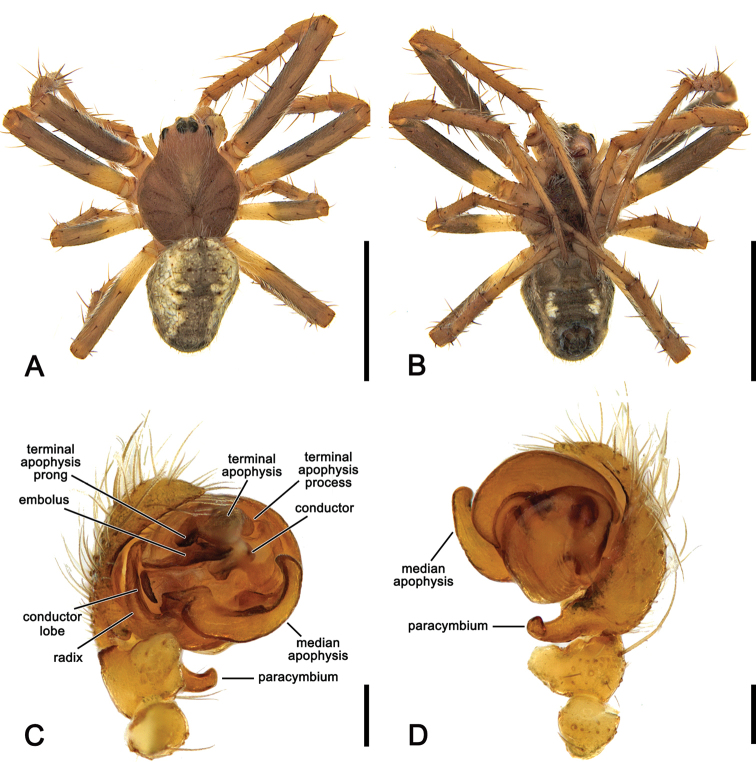
*Salsafuliginata* (L. Koch, 1872), comb. nov., male (MV K-14867 (CVIC 1163)) **A** dorsal habitus **B** ventral habitus **C** left pedipalp, ventral view **D** left pedipalp, dorsal view. Scale bars: 2 mm (**A, B**); 0.2 mm (**C, D**).

Male pedipalp patella with a single macroseta (e.g., Figs 2A–D, 3A–D, 4, 6C, D), except in *S.canalae* comb. nov. and *S.tartara* sp. nov. (Figs 12C, D, 22C, D); paracymbium of variable length, hook-like (e.g., Figs 6D, 9D, 12D, 17D); median apophysis C-shaped, generally with numerous tubercles in the basal arch (e.g., Figs 2A–D, 3A–D, 4, 6C); radix elongate (e.g., Figs 2A–C, 3A–D, 4, 6C); basal conductor lobe conspicuous, very wide anteriorly (e.g., Figs 6C, 9C, 12C); terminal apophysis slightly inflated, sub-rectangular and sometimes bearing a basal prong and/or an apical process varying in length (e.g., Figs 6C, 9C, 12C); distal haematodocha sometimes with an inflated apical section, but always inconspicuous (e.g., Figs 6C, 9C, 12C); conductor inflated and bilobed with a median dent and rounded borders (e.g., Figs 2A–D, 3A–D, 4, 6C); embolus compact and short, generally hidden by terminal apophysis in ventral view (e.g., Figs 2A–D, 3A–D, 4, 6C).

**Figure 7.**
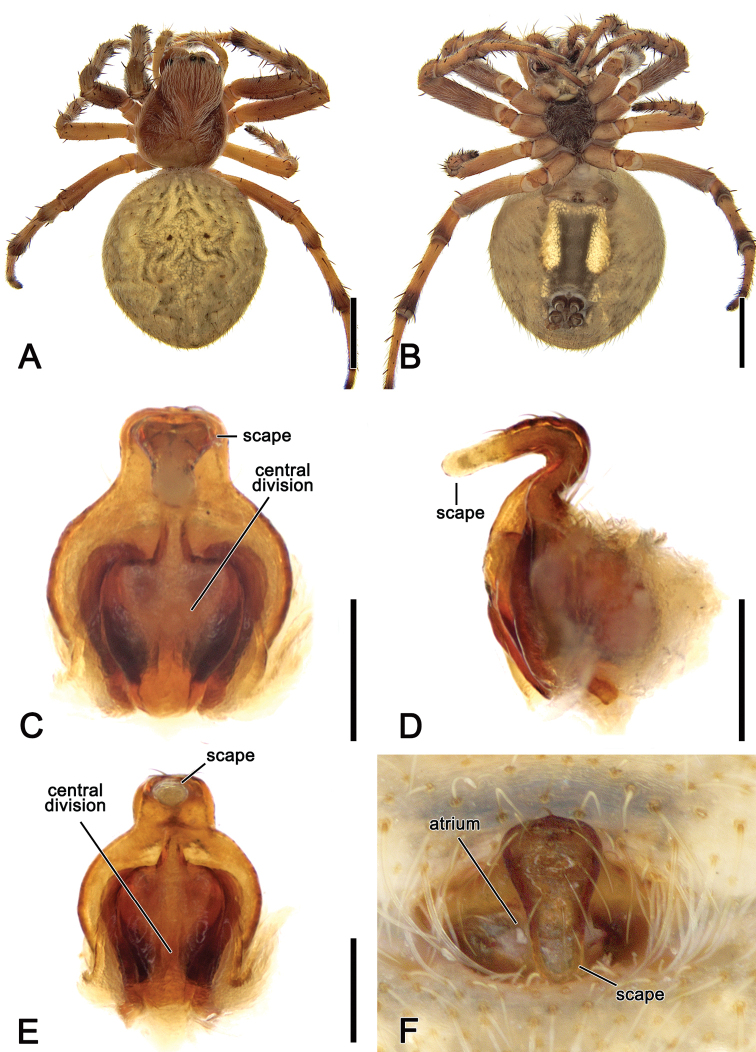
*Salsafuliginata* (L. Koch, 1872), comb. nov., female (MV K-14863 (CVIC 1173)) **A** dorsal habitus **B** ventral habitus **C** epigyne, postero-ventral view **D** epigyne, lateral view **E** epigyne, posterior view **F** epigyne in situ, ventral view (MV K-4866). Scale bars: 2 mm (**A, B**); 0.2 mm (**C–E**).

Epigyne base oval (rectangular in *S.rueda* sp. nov.), partially to strongly sclerotised with very wide atrium and central division, sometimes bearing a conspicuous ridge (e.g., Figs 7C, E, F, 10C, 13C, D); scape with wide base, transparent and generally curved apically, without or with just a few short setae, and in all but *S.canalae* comb. nov. shorter than the epigyne length (e.g., Figs 7C, E, F, 10C, 13C, D); spermathecae ovoid to spherical and very wide (Fig. 5A–F).

###### Included species.

See Table 1.

###### Distribution.

**Figure 8.**
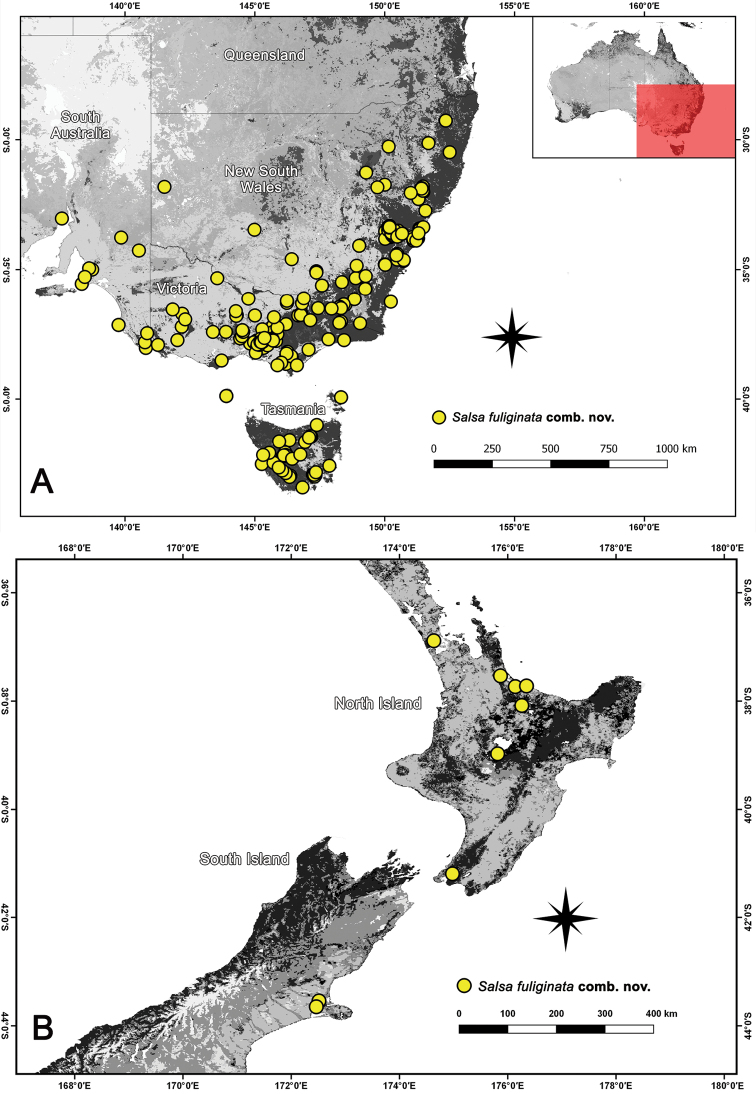
Distribution records of *Salsafuliginata* (L. Koch, 1872), comb. nov. in Australia.

*Salsa* gen. nov. is mostly known from Australia. However, *S.canalae* comb. nov. occurs only in New Caledonia, *S.neneba* sp. nov. only in Papua New Guinea, and *S.fuliginata* comb. nov. can also be found in New Zealand (Figs 8; 11; 14; 16; 21).

### ﻿Males (male of *S.neneba* sp. nov. unknown)

**Table:** 

1	Pedipalp patella with two setae (Fig. 12A, 22D)	**2**
–	Pedipalp patella with one seta (e.g., Fig. 9D)	**3**
2	Abdomen with a pointed posterior end (Fig. 12A, B); pedipalp terminal apophysis with finger-like basal prong (Fig. 12C); only known from New Caledonia (Fig. 14)	***S.canalae* comb. nov.**
–	Abdomen with a rounded posterior end (Fig. 22A, B); pedipalp terminal apophysis without a basal prong (Fig. 22C); endemic to Lord Howe Island (Fig. 21)	***S.tartara* sp. nov.**
3	Median apophysis elongate, reaching past the tegulum’s apical portion in dorsal view (Fig. 19D); terminal apophysis with a strong and elongated basal prong (Figs 3A–C, 19C)	***S.rueda* sp. nov.**
–	Median apophysis short, not reaching past the tegulum’s apical portion; terminal apophysis basal prong short and not conspicuous (e.g., Fig. 2A, B)	**4**
4	Median apophysis with acute heavily sclerotised apical tip that points basally (Fig. 9C)	***S.brisbanae* comb. nov.**
–	Median apophysis tip blunt (Figs 6C, 17C)	**5**
5	Median apophysis elongate, apically pointing towards bulb; terminal apophysis apical process blunt and rounded (Figs 2A–C; 4, 6C)	***S.fuliginata* comb. nov.**
–	Pedipalp median apophysis short, not apically pointing towards bulb; terminal apophysis apical process very strong with a pointed tip (Fig. 17C)	***S.recherchensis* comb. nov.**

### ﻿Females

**Table:** 

1	Epigyne scape reaching past the posterior edge of the epigyne base (Fig. 13C–E); only known from New Caledonia (Fig. 14)	***S.canalae* comb. nov.**
–	Epigyne scape not reaching past posterior edge of the epigyne base (e.g., Figs 10C; 15C; 20C)	**2**
2	Epigyne base much longer than wide in ventral view (Figs 20C, 22C)	**3**
–	Epigyne base as long as wide or only slightly longer	**4**
3	Epigyne centrally with narrow ridge (Fig. 20C)	***S.rueda* sp. nov.**
–	Epigyne centrally without narrow ridge (Fig. 22C)	***S.tartara* sp. nov.**
4	Epigyne base almost round with narrow lateral borders; atrium without transverse ridges (Fig. 10C, E)	***S.brisbanae* comb. nov.**
–	Epigyne not round but irregular or ovoid, transverse ridges often present	**5**
5	Epigyne borders sinuous antero-laterally and atrium with two transverse ridges (Fig. 15C); only known from Papua New Guinea (Fig. 16)	***S.neneba* sp. nov.**
–	Epigyne base inconspicuous (Fig. 7F) or antero-laterally not sinuous (Fig. 18C)	**6**
6	Epigyne base inconspicuous as epigyne plate is hidden in abdomen due to a rotation of the epigyne into the abdomen; heart-shaped atrium (i.e. Fig. 7C) not exposed (Fig. 7F); scape generally intact	***S.fuliginata* comb. nov.**
–	Epigyne conspicuous with heart-shaped atrium exposed (Fig. 18C); scape generally broken off	***S.recherchensis* comb. nov.**

#### ﻿ Salsa fuliginata

Taxon classificationAnimaliaAraneaeAraneidae

(L. Koch, 1871) comb. nov.

E0036668-B048-5FCC-A20C-6786DACEF472

Figs 1A–D , 2A–D , 4 , 5A , 6A–D , 7A–F , 8

Epeira fuliginata Koch 1872: 106–107, plate 8, fig. 7, 7a, 7b.
Epeira rubicundula Keyserling 1887: 164–165, pl. 14, fig. 1, a, b. Syn. nov.
Araneus fuliginatus (L. Koch): Simon 1895: 804; Hogg 1900: 74; Rainbow 1911: 186; Bonnet 1955: 505.
Araneus rubicundulus (Keyserling): Rainbow 1911: 192.
Cyclosa fuliginata (L. Koch): Dondale 1966: 1162–1164, fig. 3G–J.

##### Type specimen.

***Holotype*** female, Sydney (33°52'S, 151°13'E, New South Wales, Australia) (NHMW-Zoo-Ar-29914). Photographs examined.

***Holotype*** of *Epeirarubicundula* Keyserling, 1887, female, Sydney (3°53'S, 151°13'E, New South Wales, Australia). Depository unknown, not examined (see Remarks).

##### Other material examined.

162 males, 509 females (8 with egg sac), 105 juveniles (in 360 records) (see Suppl. material 1)

##### Diagnosis.

The genital morphology of males of *S.fuliginata* comb. nov. is most similar to that of *S.recherchensis* comb. nov., however, the median apopohysis is relatively longer and more slender in *S.fuliginata* comb. nov. and the terminal apophysis lacks the distinct spine-like prong present in *S.recherchensis* comb. nov. (Fig. 6C vs. Fig. 17C). The epigyne of female *S.fuliginata* comb. nov. is most similar to that of S. *recherchensis* comb. nov., but in S. *fuliginata* comb. nov. the atrium is not visible due to a rotation of the epigyne into the abdomen (Fig. 7F), whereas the atrium is visible ventrally in *S.recherchensis* comb. nov. (Fig. 18C). In addition, the apical section of the scape is straight in lateral view in *S.fuliginata* comb. nov. (Fig. 7D), but curved in *S.recherchensis* comb. nov. (Fig. 18G).

##### Redescription.

**Male** (based on MV K-14867 (CVIC 1163)): Total length 3.2. Carapace 1.8 long, 1.4 wide, dark orange-brown, with dark streaks from fovea and yellow setae throughout (Fig. 6A). Eye diameter AME 0.13, ALE 0.11, PME 0.07, PLE 0.07; row of eyes: AME 0.34, PME 0.32, PLE 0.83. Chelicerae orange-brown; with three promarginal teeth (median largest) and three retromarginal teeth (basal largest). Legs brown, femora basally, trochanters and coxae yellow-brown (Fig. 6A, B). Leg formula I > II > IV > III; length of segments (femur + patella + tibia + metatarsus + tarsus = total length): I – 2.2 + 0.9 + 1.6 + 1.7 + 0.7 = 7.1, II – 1.9 + 0.7 + 1.3 + 1.4 + 0.6 = 5.9, III – 1.2 + 0.4 + 0.7 + 0.8 + 0.4 = 3.5, IV – 1.7 + 0.6 + 1.0 + 1.1 + 0.6 = 5.0. Labium 0.22 long, 0.36 wide (Fig. 6B). Sternum 0.9 long, 0.7 wide and brown (Fig. 6B). Abdomen 1.6 long, 1.3 wide, dorsum beige with olive-grey irregular large folium, laterally dark brown mottled in black (Fig. 6A); venter dark brown with two elongate longitudinal white patches behind epigastric furrow (Fig. 6B). Pedipalp (Figs 2A–D, 4, 6C, D): length of segments (femur + patella + tibia + cymbium = total length): 0.4 + 0.2 + 0.2 + 0.5 = 1.3; paracymbium short and slightly curved; median apophysis basally pronounced with a reduced basal process, elongated and C-shaped with a blunt tip; conductor lobe robust, connecting to conductor basally of embolus; terminal apophysis sub-rectangular, bearing a thumb-like projection apically; conductor flat, poorly sclerotised; embolus elongate, pronounced and straight.

**Female** (based on MV K-14863 (CVIC1173)): Total length 9.0. Carapace 3.5 long, 2.7 wide; with colour as in male and covered by yellow setae (Fig. 7A). Eye diameter AME 0.18, ALE 0.16, PME 0.13, PLE 0.13; row of eyes: AME 0.50, PME 0.49, PLE 1.73. Chelicerae orange-brown, three promarginal teeth (median largest) and three retromarginal teeth of similar size. Legs orange-brown mottled in pale brown (Fig. 7A, B). Pedipalp length of segments (femur + patella + tibia + tarsus = total length): 1.0 + 0.5 + 0.6 + 1.1 = 3.2. Leg formula I > II > IV > III; length of segments (femur + patella + tibia + metatarsus + tarsus = total length): I – 3.5 + 1.6 + 2.9 + 2.9 + 1.1 = 12.0, II – 3.2 + 1.4 + 2.4 + 2.5 + 1.0 = 10.5, III – 2.0 + 1.0 + 1.2 + 1.2 + 0.7 = 6.1, IV – 3.1 + 1.3 + 2.2 + 2.4 + 0.8 = 9.8. Labium 0.49 long, 0.72 wide, dark brown; endites dark brown to brown (Fig. 7B). Sternum 1.6 long, 1.5 wide, dark brown with grey setae (Fig. 7B). Abdomen 5.4 long, 4.9 wide; dorsum and laterally olive-grey with dorsal darker folium pattern (Fig. 7A); venter dark olive-grey with lateral elongate ovoid pale bands connected with pale band behind epigastric furrow (Fig. 7B). Epigyne wider than long in ventral view (Fig. 7F); atrium/central division heart-shaped (Fig. 7C, E); scape elongate sub-triangular (Fig. 7C, F); spermathecae spherical and very large (Fig. 5A).

##### Variation.

Total length males 3.2–5.5 (*n* = 7); females 4.5–9.2 (*n* = 10). As in many orb-weaving spiders, colour patterns in *S.fuliginata* comb. nov. can vary considerably, mainly in how distinct the folium is and how well it is delineated. Colour shades range from pale beige to orange- and reddish brown to dark brown (e.g., Fig. 1A–D).

##### Remarks.

Renner (1988) listed a ‘cotyp’ in the Stuttgart Museum that was destroyed in WWII. However, the original description clearly states (L. Koch 1872, p. 107: “Von Sydney. Ein Exemplar im k. k. Museum zu Wien” (= From Sydney. One Specimen in the Vienna Museum), which means the female specimen present in the NHMW should be considered the single holotype and the specimen destroyed in the Stuttgart Museum was not of taxonomic relevance.

Rack (1961) listed a female collected in Sydney as holotype of *Epeirarubicundula* from the ZMH (Rack (1961)-catalogue no. 270). This specimen, however, does not match the original description by Keyserling (1887), but is clearly a female of *S.brisbanae* comb. nov. Keyserling’s (1887) illustration of the female clearly shows the small sub-triangular scape of *S.fuliginata* comb. nov. with a very small epigyne plate, also typical for that species. We could not find a female specimen in any historical collection (NHMUK, ZMH, ZMB) that matched the description of *E.rubicundula* and was labelled as such. We therefore consider the holotype of this species lost. The original description, however, leaves no doubt that this species is conspecific with *S.fuliginata* comb. nov. and we therefore consider *E.rubicundula* a junior synonym of *S.fuliginata* comb. nov.

##### Life history and habitat preferences.

Mature males of *S.fuliginata* comb. nov. are more common from October to January, with much lower numbers occurring in other months, especially September and February. Very few adults were found between these two months, with no males recorded for August. Equally, females are spring/summer-mature, with the majority of specimens being collected between October and January, but with comparatively more specimens in September and February than males. Few females have been collected between February and September, but differently from males there are no specimens collected in July and one that was collected in August.

*Salsafuliginata* comb. nov. does not seem to be very habitat-specific as it has been found in a variety of forests to more open habitats with lower vegetation. Habitat descriptions on labels with specimens include “dry sclerophyll forest”, “open forest”, “shrubs”, and “bushes”; but also “garden” and “swamps”.

##### Distribution.

The distribution of *S.fuliginata* comb. nov. encompasses four Australian states: New South Wales, South Australia, Tasmania, and Victoria (Fig. 8A). This species is also found in New Zealand (Fig. 8B).

#### ﻿ Salsa brisbanae

Taxon classificationAnimaliaAraneaeAraneidae

(L. Koch, 1867) comb. nov.

564ED121-33E4-5DD0-8142-A4EC56E807A3

**Figure 9.**
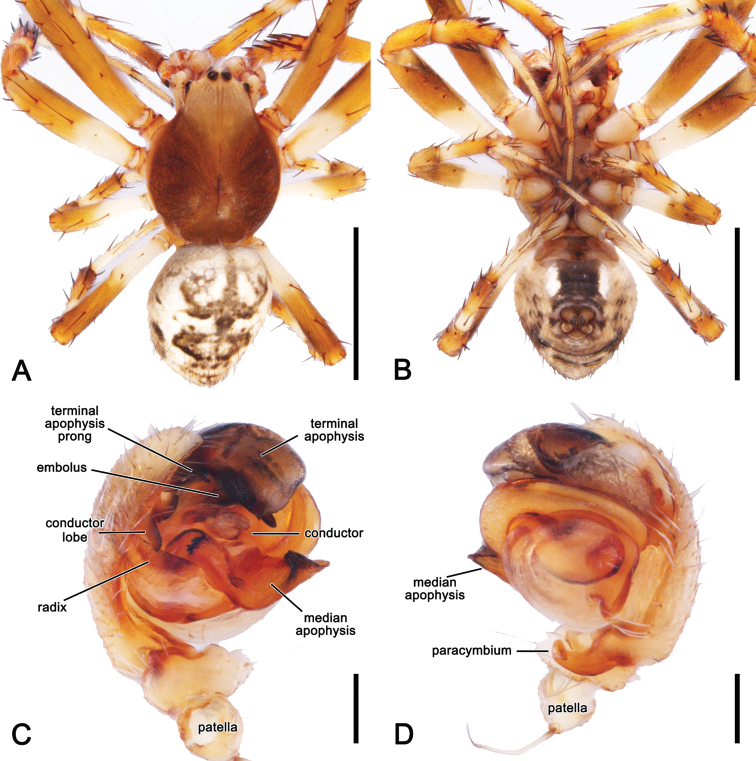
*Salsabrisbanae* (L. Koch, 1867), comb. nov., male (NHMD 12231) **A** dorsal habitus **B** ventral habitus **C** left pedipalp, ventral view **D** left pedipalp, dorsal view. Scale bars: 2 mm (**A, B**); 0.2 mm (**C, D**).

**Figure 10.**
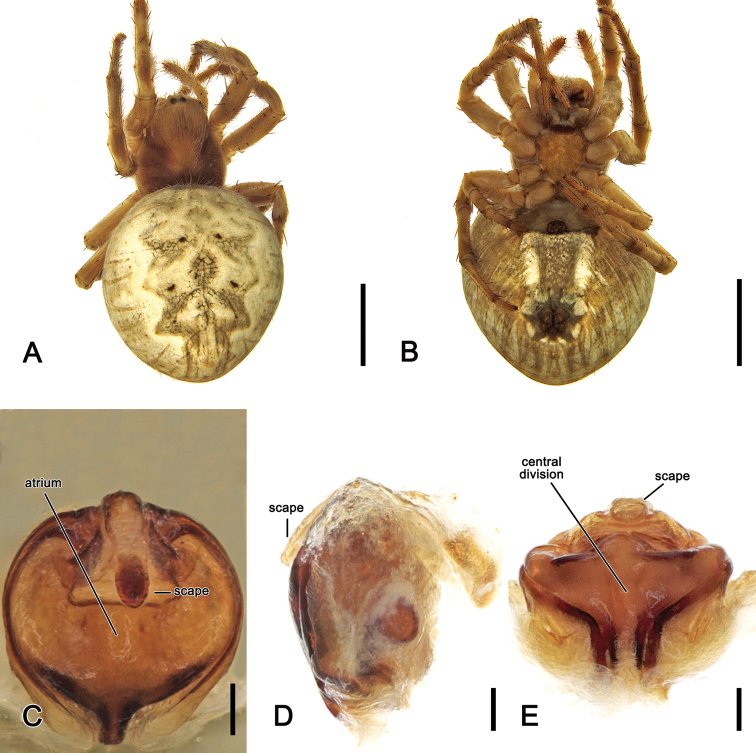
*Salsabrisbanae* (L. Koch, 1867), comb. nov., female (AM KS.131087) **A** dorsal habitus **B** ventral habitus **C** epigyne, ventral view **D** epigyne, lateral view **E** epigyne, posterior view. Scale bars: 2 mm (**A, B**); 0.2 mm (**C–E**).

**Figure 11.**
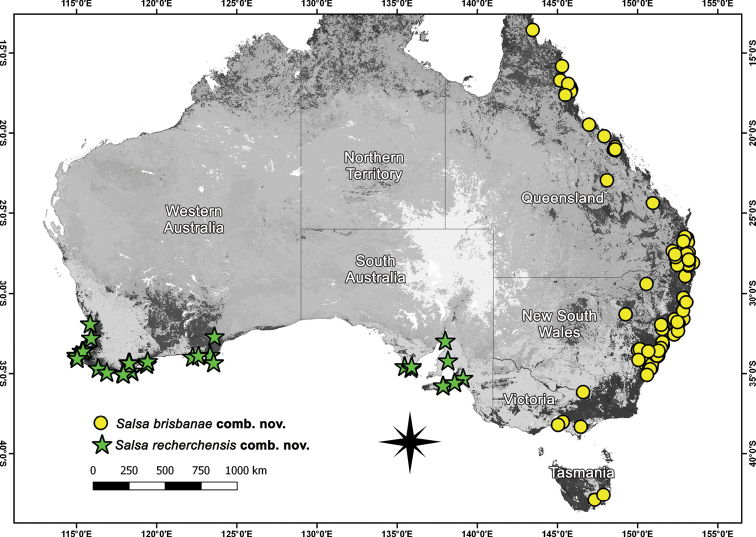
Distribution records of *Salsabrisbanae* (L. Koch, 1867), comb. nov. and *Salsarecherchensis* (Main, 1954), comb. nov. in Australia.

Figs 5B , 9A–D , 10A–E , 11

Epeira brisbanae Koch 1867: 176–177; Koch 1872: 111–112, plate 6, fig. 4; Keyserling 1887: 161–164, plate 13, figs 6, 6a–d, 7, 7a.
Araneus brisbanae (L. Koch): Simon 1895: 804; Rainbow 1911: 183; Dalmas 1917: 387–388.
Araneus brisbanensis (L. Koch): Bonnet 1955: 448.

##### Type specimen.

***Holotype*** of *Epeirabrisbanae* L. Koch, 1872, female, Brisbane (27°28'S, 153°01'E, Queensland, Australia) (ZMH (Rack 1961)-catalogue no. 226). Examined.

##### Other material examined.

57 males, 208 females (2 with egg sac), 39 juveniles (in 146 records) (see Suppl. material 1).

##### Diagnosis.

Male *S.brisbanae* comb. nov. can be distinguished from all other *Salsa* gen. nov. species by the unique morphology of the pedipalp median apophysis that has a very acute, basally pointed tip (Fig. 9C) (median apophysis generally rounded C-shaped in all other species). Female genitalia are probably most similar to those of S. *canalae* comb. nov., but the scape of *S.brisbanae* comb. nov. is shorter than the epigyne plate (Fig. 10C), whereas it is longer than the plate in *S.canalae* comb. nov. (Fig. 13C, D).

##### Redescription.

**Male** (based on NHMD 12231). Total length 4.4. Carapace 2.3 long, 1.9 wide, dark brown, slightly paler anteriorly (Fig. 9A). Eye diameter AME 0.12, ALE 0.11, PME 0.14, PLE 0.09; row of eyes: AME 0.32, PME 0.33, PLE 0.90. Chelicerae pale brown; with three promarginal teeth (median largest) and three retromarginal teeth (basal largest). Legs brown, femora basally yellow-brown (Fig. 9A, B). Leg formula I > II > IV > III; length of segments (femur + patella + tibia + metatarsus + tarsus = total length): I – 2.5 + 1.1 + 2.0 + 1.6 + 0.7 = 7.9, II – 2.2 + 1.0 + 1.4 + 1.5 + 0.6 = 6.7, III – 1.4 + 0.6 + 0.7 + 0.7 + 0.5 = 3.9, IV – 2.0 + 0.7 + 1.3 + 1.4 + 0.6 = 6.0. Labium 0.27 long, 0.35 wide, brown; endites beige (Fig. 9B). Sternum 1.1 long, 0.7 wide, dark brown with black radial shading (Fig. 9B). Abdomen 2.1 long, 1.7 wide, posteriorly pointed; dorsum with beige background and large, irregular, olive-grey, folium, laterally pale olive-grey with black streaks (Fig. 9A); venter dark grey, laterally with two elongate white bands (Fig. 9B). Pedipalp (Fig. 9C, D) length of segments (femur + patella + tibia + cymbium = total length): 0.3 + 0.2 + 0.1 + 0.65 = 1.25; paracymbium strong and curved apically; median apophysis transverse, terminating in an acute and basally pointed tip; denticles in basal arch of median apophysis distinct; conductor lobe narrow; terminal apophysis enlarged, sub-rectangular, bearing a reduced basal prong; conductor bilobed; embolus short, heavily sclerotised.

**Female** (based on AM KS.131087): Total length 6.9. Carapace 3.0 long, 2.3 wide; dark brown, cephalic area paler (Fig. 10A). Eye diameter AME 0.14, ALE 0.09, PME 0.07, PLE 0.07; row of eyes: AME 0.41, PME 0.38, PLE 1.35. Chelicerae orange-brown, four promarginal teeth (apical and second basal largest) and three retromarginal teeth of similar size. Legs brown, patellae and tibiae apically slightly darker (Fig. 10A, B). Pedipalp length of segments (femur + patella + tibia + tarsus = total length): 0.9 + 0.5 + 0.5 + 1.0 = 2.9. Leg formula I > II > IV > III; length of segments (femur + patella + tibia + metatarsus + tarsus = total length): I – 2.4 + 1.1 + 1.8 + 1.9 + 0.9 = 8.1, II – 2.1 + 1.1 + 1.5 + 1.7 + 0.7 = 7.1, III – 1.2 + 0.7 + 0.8 + 0.8 + 0.5 = 4.0, IV – 2.1 + 1.0 + 1.4 + 1.5 + 0.6 = 6.6. Labium 0.36 long, 0.59 wide, dark brown; endites dark brown (Fig. 10B). Sternum 1.3 long, 1.1 wide, orange-brown, with some paler discolourations (Fig. 10B). Abdomen 4.7 long, 4.6 wide; dorsum beige with olive-brown folium, laterally covered by orange-brown streaks (Fig. 10A); venter olive-grey centrally with paler mottles, laterally with elongate white bands (Fig. 10B). Epigyne base almost circular, slightly wider than long, with narrow elevated borders and therefore atrium extends almost over whole base (Fig. 10C); scape slightly less than half of epigyne base, slightly wrinkled and its sides parallel (Fig. 10C); central division wide and abruptly tapering dorsally; spermathecae narrow pointing apically (Fig. 5B).

##### Variation.

Only one other male was measured, total length 3.9; females total lengths 6.9–7.9 (*n* = 4). Like in other species of the genus, the colour variations in *S.brisbanae* comb. nov. can be considerable and range from pale to dark brown tones in live specimens with the folium pattern on the abdomen more or less distinct.

##### Life history and habitat preferences.

Male and female specimens of *S.brisbanae* comb. nov. have mainly been found between October and May, with only few specimens collected from June to September. Although mature spiders can therefore be found all year round, the species should be considered as mainly late-summer to autumn mature. Most specimens were apparently collected on leaves and bark as labels indicate sweeping and beating as the main collection techniques that were used to capture the spiders.

*Salsabrisbanae* comb. nov. does not seem to be very habitat-specific, with specimens being collected in completely different environments, mostly in lower vegetation. Habitat descriptions on labels with specimens include “rainforest”, “shrubs”, “grass”, and “foliage”; but also “dune” and “lagoon vegetation”. Plant species that were cited at collection sites include *Acacialongifolia* (long-leaved wattle), *Leptospermumlaevigatum* (coast tea tree), and *Monotocaelliptica* (tree broom heath).

##### Distribution.

*Salsabrisbanae* comb. nov. occurs in Queensland, New South Wales, Victoria and Tasmania (Fig. 11). The species was recorded by Keyserling (1887) from New Zealand (see also Dalmas 1917), but this record is likely based on a misidentification (Paquin et al. 2008). The species was apparently also recorded from Papua New Guinea (World Spider Catalog 2022), but we could not find any original citation that suggests this distribution.

#### ﻿ Salsa canalae

Taxon classificationAnimaliaAraneaeAraneidae

(Berland, 1924) comb. nov.

34549E3D-7219-5085-ACAA-DFFD56D02392

**Figure 12.**
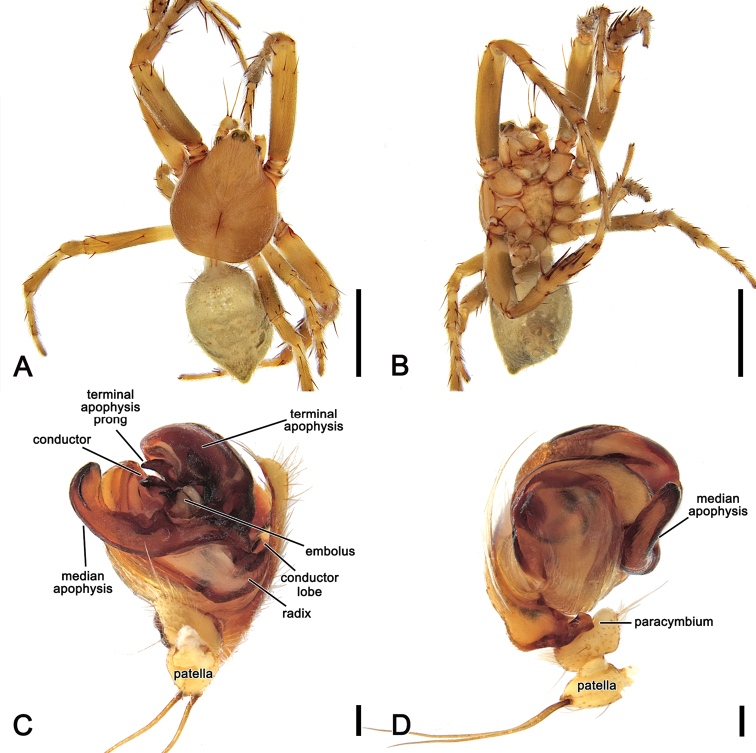
*Salsacanalae* Berland, 1924, comb. nov., male (WAM T75922) **A** dorsal habitus **B** ventral habitus **C** left pedipalp, ventral view **D** left pedipalp, dorsal view. Scale bars: 2 mm (**A, B**); 0.2 mm (**C, D**).

**Figure 13.**
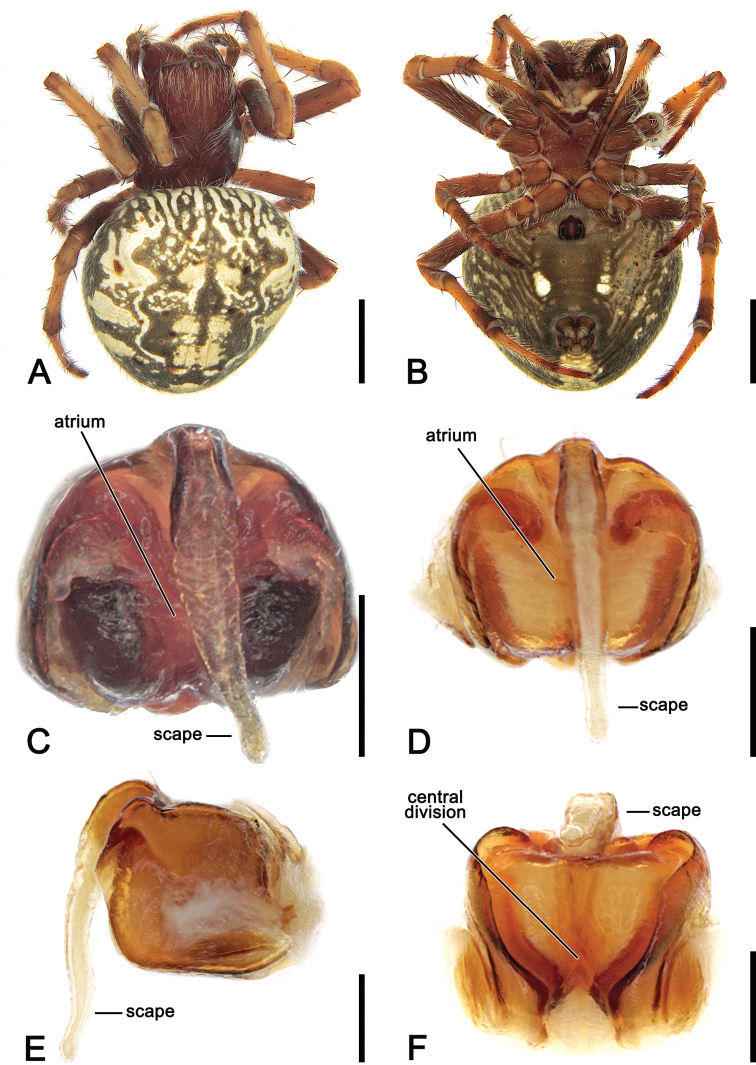
*Salsacanalae* Berland, 1924, comb. nov., female **A** dorsal habitus (WAM T75921) **B** ventral habitus (WAM T75921) **C** epigyne, ventral view (WAM T75921) **D** epigyne variation, ventral view (WAM T75923) **E** epigyne variation, lateral view (WAM T75923) **F** epigyne variation, posterior view (WAM T75923). Scale bars: 2 mm (**A, B**); 0.2 mm (**C–F**).

**Figure 14.**
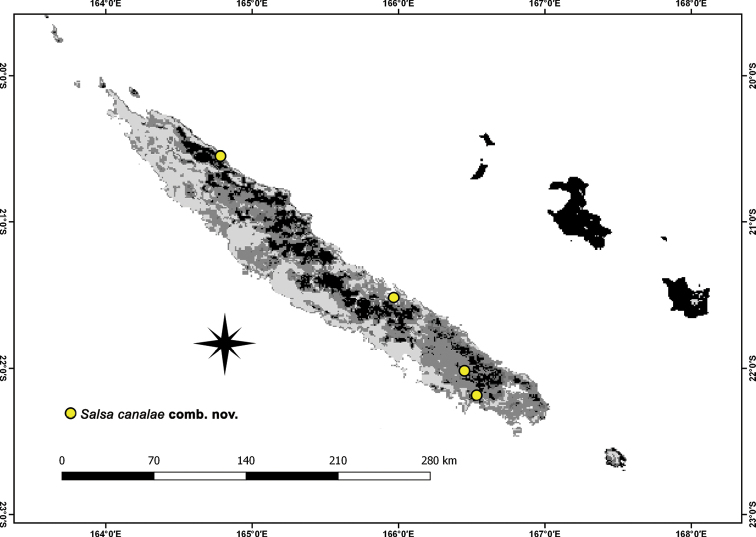
Distribution records of *Salsacanalae* Berland, 1924, comb. nov. in New Caledonia.

Figs 5C , 12A–D , 13A–F , 14

Araneus canalae Berland 1924: 222, fig. 126, 127; Berland 1931: 666; Berland 1932: 296, 298–299.
Araneus canalensis Berland. Bonnet 1955: 459.

##### Type specimen.

***Holotype*** female, Mount Canala (21°31'S, 165°58'E, New Cale­donia), F. Sarasin and J. Roux (NHMB 979a). Photographs examined.

##### Other material examined.

1 male, 7 females (in 8 records)(see Suppl. material 1).

##### Diagnosis.

Males of *S.canalae* comb nov. shares with *S.tartara* sp. nov. two patellar setae on the pedipalp (Fig. 12C, D vs. Fig. 22C, D). However, *S.canalae* comb nov. is distinguished by prominent, heavily sclerotised conductor of *S.tartara* sp. nov. which is short and inconspicuous in *S.canalae* comb. nov. Female genitalia are most similar to those of *S.brisbanae* comb. nov., but differ from those and other *Salsa* gen. nov. species by the epigyne scape, that is longer than the epigyne plate and exceeds its posterior margin (Fig. 13C, D).

##### Redescription.

**Male** (based on WAM T75922) Total length 5.5. Carapace 2.9 long, 2.5 wide, pear-shaped and pale brown, covered with short white setae (Fig. 12A). Eye diameter AME 0.20, ALE 0.18, PME 0.13, PLE 0.11; row of eyes: AME 0.54, PME 0.47, PLE 1.22. Chelicerae yellowish brown; with four promarginal teeth (second basal largest) and three retromarginal teeth (basal largest). Legs yellowish brown mottled in pale brown, bearing thick setae on patella, tibia and metatarsus (Fig. 12A, B). Leg formula I > II > IV > III; length of segments (femur + patella + tibia + metatarsus + tarsus = total length): I – 2.8 + 1.2 + 2.2 + 1.7 + 0.9 = 8.8, II – 2.2 + 1.0 + 1.6 + 1.6 + 0.8 = 7.2, III – 1.4 + 0.6 + 0.8 + 0.8 + 0.5 = 4.1, IV – 2.1 + 0.9 + 1.5 + 1.5 + 0.7 = 6.7. Labium 0.31 long, 0.47, and endites yellowish brown, paler anteriorly (Fig. 12B). Sternum 1.3 long, 0.8 wide, yellowish brown mottled dark and bearing dark brown contour (Fig. 12B). Abdomen 2.5 long, 1.8 wide, with pointed conical posterior portion after spinnerets, dorsum, sides, and venter beige mottled in grey (Fig. 12A, B). Pedipalp (Fig. 12C, D) length of segments (femur + patella + tibia + cymbium = total length): 0.5 + 0.2 + 0.15 + 0.9 = 1.75; patella with two setae; paracymbium reduced and straight; median apophysis elongated, with a thick basal process, a notched apical section on an acute and apically curved rounded tip; conductor lobe small; terminal apophysis subrectangular, apically projected and inflated, bearing a finger-like lobe from its basis; conductor flat with sclerotised borders; embolus short and strong, very sclerotised.

**Female** (based on WAM T75921): Total length 8.3. Carapace 3.5 long, 3.1 wide; dark brown and bearing long white setae throughout (Fig. 13A). Eye diameter AME 0.20, ALE 0.18, PME 0.14, PLE 0.13; row of eyes: AME 0.52, PME 0.45, PLE 1.88. Chelicerae dark brown, four promarginal teeth (apical and basal largest), and three retromarginal (basal largest). Legs orange-brown, slightly darker on femur and joints (Fig. 13A, B). Pedipalp length of segments (femur + patella + tibia + tarsus = total length): 1.0 + 0.5 + 0.7 +1.1 = 3.3. Leg formula I > II > IV > III; length of segments (femur + patella + tibia + metatarsus + tarsus = total length): I – 3.2 + 1.4 + 2.7 + 2.4 + 1.1 = 10.8, II – 2.8 + 1.5 + 2.2 + 2.2 + 0.9 = 9.6, III – 1.9 + 0.9 + 1.0 + 1.0 + 0.7 = 5.5, IV – 2.8 + 1.2 + 2.0 + 2.1 + 0.9 = 9.0. Labium 0.54 long, 0.86 wide and endites dark brown, beige on anterior border (Fig. 13B). Sternum 1.5 long, 1.5 wide and brown (Fig. 13B). Abdomen 5.5 long, 5.2 wide; dorsum with beige background brindled in olive-grey (Fig. 13A); sides olive-grey (Fig. 13B); venter olive-grey with two thick rounded white patches (Fig. 13B). Epigyne subquadrate with broadly rounded antero-lateral borders and wide atrium and basis (Fig. 13C, D); scape almost twice the length of epigyne base, from a slightly wide base gradually narrowing a thin section (Fig. 13C–E); central division goblet-like, very wide anteriorly, ca. as wide as the epigyne base, and tapering basally (Fig. 13F); spermathecae oval and apart by more than its diameter (Fig. 5C).

##### Variation.

Only one male was available for measurements (see above); female total lengths 5.8 and 6.4 (*n* = 2). All our specimens were of very similar colouration, but Berland (1932) reported numerous females with considerable colour variations, specifically of the abdomen, without providing any further detail.

##### Life history and habitat preferences.

The mature male described here was found in April, mature females examined between February and June; however, specimen numbers are too small to confidently interpret the phenology of the species. There was no information on habitat with any specimen labels.

##### Distribution.

Distributed throughout New Caledonia (Fig. 14), including Nouméa (cited in Berland 1932).

#### ﻿ Salsa neneba sp. nov.

Taxon classificationAnimaliaAraneaeAraneidae

522C98A1-F781-5188-8BD6-67AC3ED092B6

http://zoobank.org/BB329767-2803-47B2-9EF1-EDD0D35CE775

Figs 15A–C , 16

##### Type specimen.

***Holotype*** female, Neneba (8°45'S, 147°30'E, Papua New Guinea), 9 November 1896 (QM S111920).

##### Other material examined.

Only known from holotype.

##### Etymology.

The specific epithet is a noun in apposition referring to the type locality.

##### Diagnosis.

**Figure 15.**
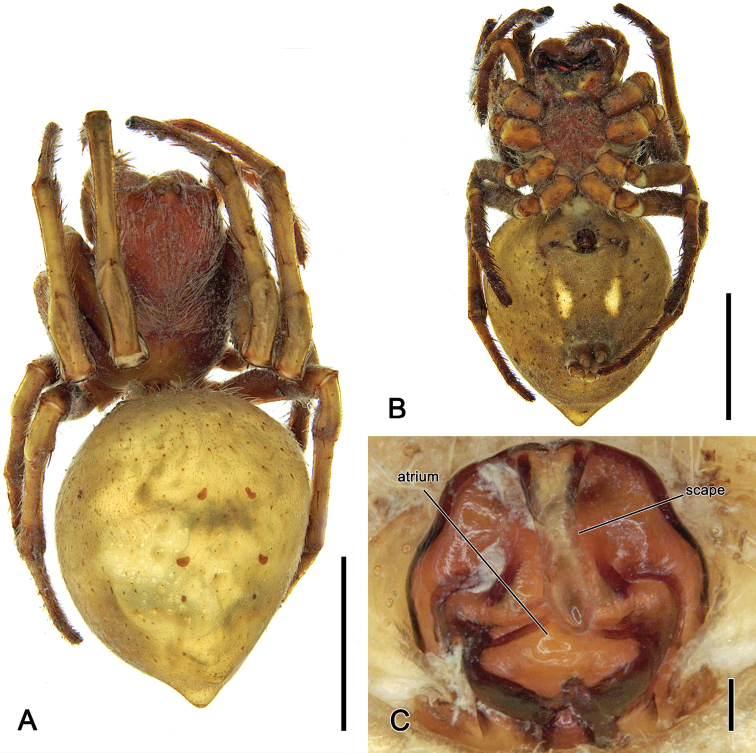
*Salsaneneba* sp. nov., female holotype (QM S111920) **A** dorsal habitus **B** ventral habitus **C** epigyne, ventral view. Scale bars: 5 mm (**A, B**); 0.1 mm (**C**).

Males of *S.neneba* sp. nov. are unknown. Genitalia of female specimens of *S.neneba* sp. nov. can be recognised by the somewhat sinuous antero-lateral edges of the epigyne plate and the transverse edges in the atrium (Fig. 15C).

##### Description.

**Male.** Unknown.

**Female** (based on holotype, QM S111920): Total length 10.5. Carapace 4.5 long, 3.2 wide; dark reddish brown and covered by white setae anteriorly from fovea (Fig. 15A). Eye diameter AME 0.23, ALE 0.18, PME 0.20, PLE 0.16; row of eyes: AME 0.68, PME 0.56, PLE 2.25. Chelicerae reddish brown, four promarginal teeth (apical and second basal largest) and three retromarginal teeth (apical smallest). Legs yellowish brown, femora slightly darker (Fig. 15A, B). Pedipalp length of segments (femur + patella + tibia + tarsus = total length): 1.2 + 0.5 + 0.8 + 1.1 = 3.6. Leg formula I > II > IV > III; length of segments (femur + patella + tibia + metatarsus + tarsus = total length): I – 3.8 + 1.7 + 3.2 + 2.8 + 1.0 = 12.5, II – 3.5 + 1.6 + 2.6 + 2.6 + 0.9 = 11.2, III – 2.1 + 1.0 + 1.2 + 1.3 + 0.7 = 6.3, IV – 3.3 + 1.4 + 2.3 + 2.3 + 0.8 = 10.1. Labium 0.58 long, 0.77 wide, reddish brown; endites reddish brown (Fig. 15B). Sternum 2.0 long, 1.8 wide, reddish brown (Fig. 15B). Abdomen 6.1 long, 5.0 wide; posterior hump distinct (Fig. 15A, B); dorsum colouration poorly preserved, beige with indistinct greyish folium pattern (Fig. 15A); venter olive-brown with two spindle-shaped pale lateral bands (Fig. 15B). Epigyne ca. as long as wide, with sinuous antero-lateral borders and transvers ridges within the atrium (Fig. 15C); scape slightly longer than half the length of the epigyne base, slightly narrowest centrally (Fig. 15C); epigyne not dissected to investigate posterior and internal morphology as only known from fragile holotype.

##### Variation.

Only known from holotype.

##### Life history and habitat preferences.

Unknown.

##### Distribution.

**Figure 16.**
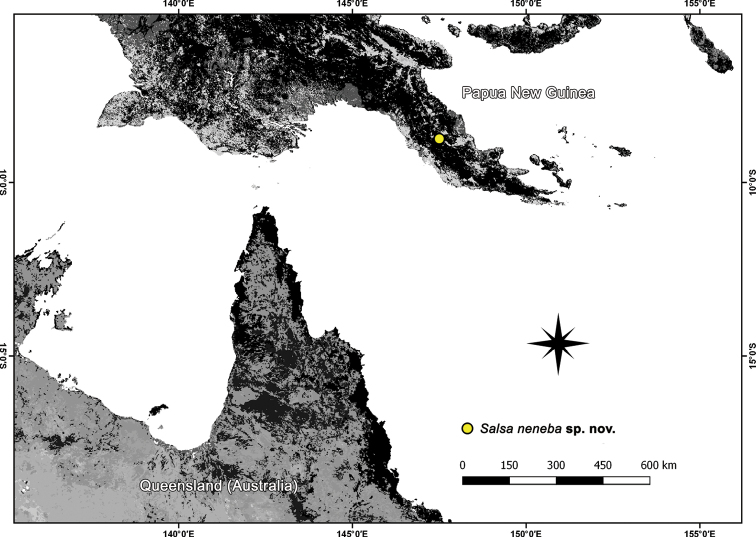
Distribution records of *Salsaneneba* sp. nov.

Currently only known from type locality, Neneba in Papua New Guinea (Fig. 16).

#### ﻿ Salsa recherchensis

Taxon classificationAnimaliaAraneaeAraneidae

(Main, 1954) comb. nov.

4F086C14-0D76-547B-98A7-3EC1F4888CFC

**Figure 17.**
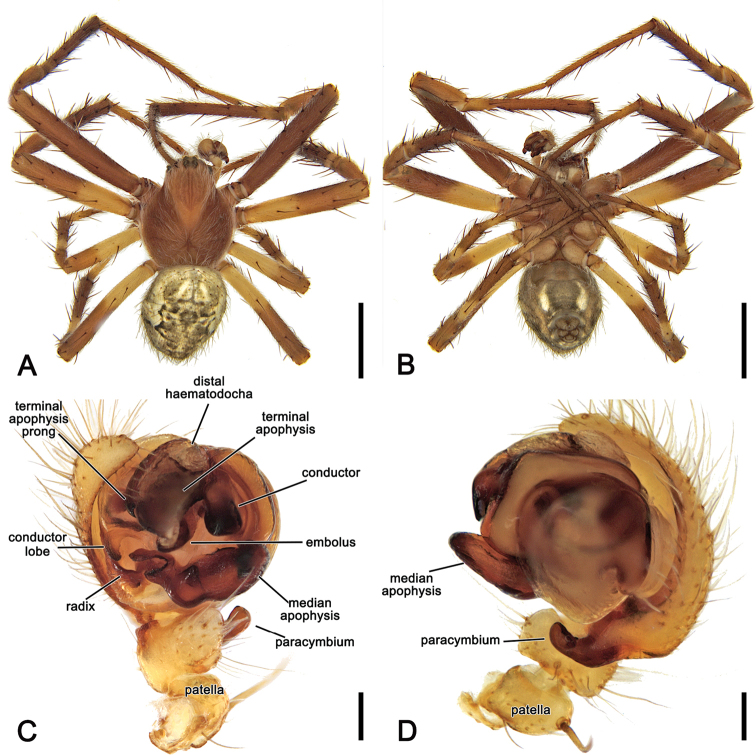
*Salsarecherchensis* (Main, 1954), comb. nov., male (WAM T77696) **A** dorsal habitus **B** ventral habitus **C** left pedipalp, ventral view **D** left pedipalp, dorsal view. Scale bars: 2 mm (**A, B**); 0.2 mm (**C, D**).

**Figure 18.**
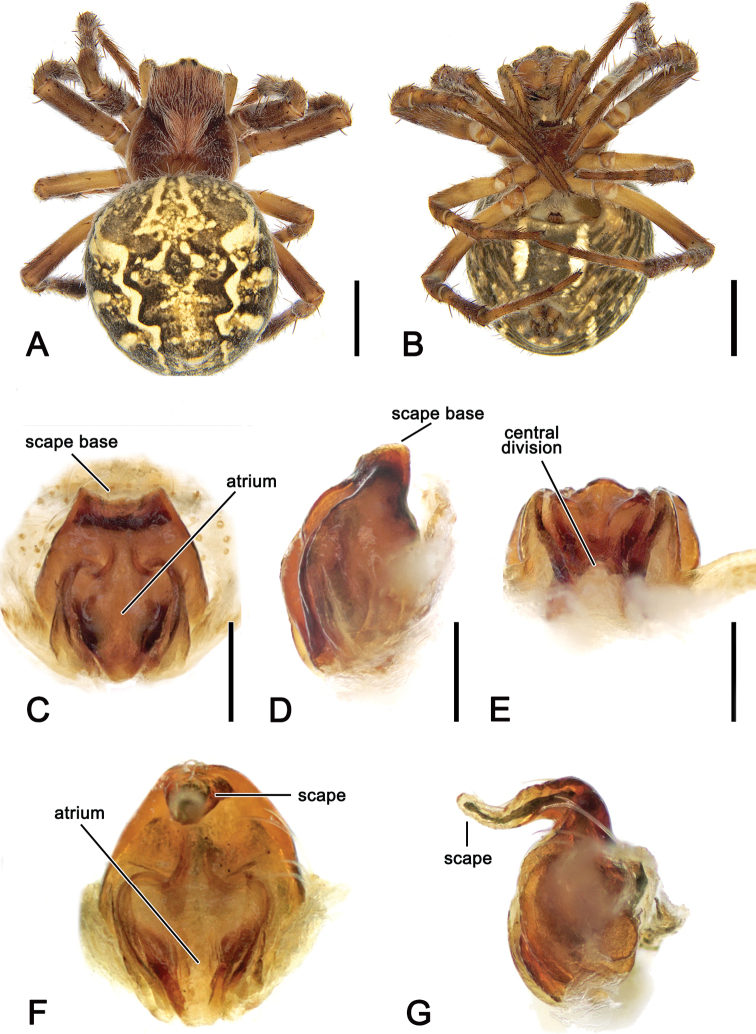
*Salsarecherchensis* (Main, 1954), comb. nov., female (WAM T77362) **A** dorsal habitus **B** ventral habitus **C** epigyne, ventral view **D** epigyne, lateral view **E** epigyne, posterior view **F** variation of epigyne, ventral view (WAM 92/2120) **G** variation of epigyne, lateral view (WAM 92/2120). Scale bars: 2 mm (**A, B**); 0.2 mm (**C–E**).

Figs 5D , 11 , 17A–D , 18A–G

Aranea recherchensis Main 1954: 41, pl. 3, figs 5, 8.

##### Type specimen.

***Holotype*** female, Figure of Eight Island, Recherche Archipelago, (34°01'S, 122°14'E, Western Australia, Australia), 7 November 1950, V. Serventy (WAM 55/4984). Examined.

##### Other material examined.

34 males, 321 females, 175 juveniles (in 74 records) (see Suppl. material 1).

##### Diagnosis.

The genital morphology of male *S.recherchensis* comb. nov. is most similar to that of *S.fuliginata* comb. nov.; however, *S.recherchensis* comb. nov. males can be distinguished by the comparatively shorter median apophysis and the distinct basal spine-like prong on the terminal apophysis (Fig. 6C vs. Fig. 17C). The epigyne of female *S.recherchensis* comb. nov. is most similar to that of *S.fuliginata* comb. nov. However, in ventral view, the epigyne plate of *S.fuliginata* comb. nov. is inconspicuous (Fig. 7F), whereas it is pronounced in *S.recherchensis* comb. nov. (Fig. 18C).

##### Redescription.

**Male** (based on WAM T73696). Total length 5.1. Carapace 2.9 long, 2.4 wide, brown, paler in cephalic area and with yellowish setae throughout (Fig. 17A). Eye diameter AME 0.18, ALE 0.14, PME 0.09, PLE 0.09; row of eyes: AME 0.50, PME 0.43, PLE 1.39. Chelicerae brown; with four promarginal teeth (second basal and apical largest) and three retromarginal teeth (basal largest). Legs brown, femora basally yellow-brown, except in leg I (Fig. 17A, B). Leg formula I > II > IV > III; length of segments (femur + patella + tibia + metatarsus + tarsus = total length): I – 4.2 + 1.6 + 3.2 + 3.3 + 1.0 = 13.3, II – 3.4 + 1.3 + 2.2 + 2.8 + 1.0 = 10.7, III – 2.2 + 0.9 + 1.1 + 1.4 + 0.7 = 6.3, IV – 3.2 + 1.1 + 2.2 + 2.5 + 0.9 = 10.0. Labium 0.34 long, 0.56 wide, brown; endites orange-brown (Fig. 17B). Sternum 1.5 long, 1.0 wide, brown (Fig. 17B). Abdomen 2.3 long, 2.1 wide, dorsum with dark grey, irregular folium on a beige background, laterally olive-grey (Fig. 17A); venter olive-grey, laterally with two elongate, curved longitudinal bands (Fig. 17B). Pedipalp length of segments (femur + patella + tibia + cymbium = total length): 0.6 + 0.2 + 0.15 + 0.8 = 1.75; paracymbium stout and slightly curved apically (Fig. 17D); median apophysis short with thick rounded tip, numerous small teeth-like tubercles inside basal arch (Fig. 17C); conductor lobe short (Fig. 17C); terminal apophysis conspicuous, sub-rectangular and bearing spine-like basal prong (Fig. 17C); conductor slightly folding over itself, broadly lapped and heavily sclerotised (Fig. 17C, D); embolus short (Fig. 17C).

**Female** (based on WAM T77362): Total length 8.0. Carapace 3.7 long, 2.7 wide; similar to male but slightly darker and more setae (Fig. 18A). Eye diameter AME 0.20, ALE 0.18, PME 0.13, PLE 0.11; row of eyes: AME 0.52, PME 0.47, PLE 1.76. Chelicerae pale brown, four promarginal teeth (apical and second basal largest) and three retromarginal teeth (basal largest). Legs pale brown mottled in dark (Fig. 18A, B). Pedipalp length of segments (femur + patella + tibia + tarsus = total length): 0.9 + 0.5 + 0.7 + 1.1 = 3.2. Leg formula I > II > IV > III; length of segments (femur + patella + tibia + metatarsus + tarsus = total length): I – 3.4 + 1.5 + 2.7 + 2.9 + 1.1 = 11.6, II – 2.8 + 1.3 + 2.3 + 2.4 + 1.0 = 9.8, III – 2.0 + 1.0 + 1.3 + 1.3 + 0.7 = 6.3, IV – 3.0 + 1.2 + 2.0 + 2.2 + 0.8 = 9.2. Labium 0.34 long, 0.72 wide, dark brown; endites dark brown (Fig. 18B). Sternum 1.5 long, 1.4 wide, dark brown (Fig. 18B). Abdomen 5.1 long, 4.7 wide; folium pattern as in male, but more distinct (Fig. 18A, B). Epigyne base slightly longer than wide; atrium heart-shaped (Fig. 18C); central division ca. as wide as the epigyne base, slightly narrowing dorsally (Fig. 18E); spermathecae spherical (Fig. 5D); scape (Fig. 18F, G.) (WAM 90/2120) broadest at base, tapering, curved in lateral view.

##### Variation.

Only a single male was measured for this study; female total length 4.5–8.0 (*n* = 6). The colour variation in this species is very similar to that of *S.fuliginata* comb. nov. and *S.brisbanae* comb. nov. with abdominal shades of beige to reddish brown and more or less conspicuous folium pattern. Of the six females measured for this study, all but one had their scapes broken off.

##### Life history and habitat preferences.

All specimens were collected between October and May, with peak collection numbers in November and January. There is not much information about habitat preferences of *Salsarecherchensis* comb. nov., but they seem to be more common in lower vegetation layers based on descriptions on specimen labels, which include “web in garden”, “understorey Karri forest”, “bushes”, “granite”, “between limestone”, and “camp”.

##### Distribution.

*Salsarecherchensis* comb. nov. is the only species of the genus found in Western Australia, although its range extends into southern South Australia (Fig. 11).

#### ﻿ Salsa rueda sp. nov.

Taxon classificationAnimaliaAraneaeAraneidae

40F65DBA-5D59-5CD4-9B2A-1BD79071F9EA

http://zoobank.org/5D907A83-BDB5-48E0-B976-0B993B9D94C2

**Figure 19.**
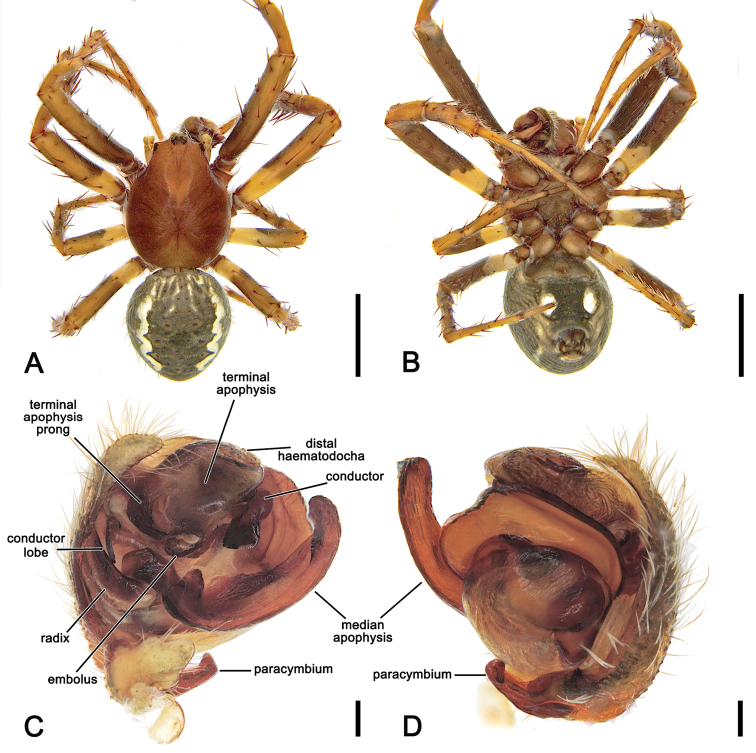
*Salsarueda* sp. nov., male holotype (MV K-14856) **A** dorsal habitus **B** ventral habitus **C** left pedipalp, ventral view **D** left pedipalp, dorsal view. Scale bars: 2 mm (**A, B**); 0.2 mm (**C, D**).

**Figure 20.**
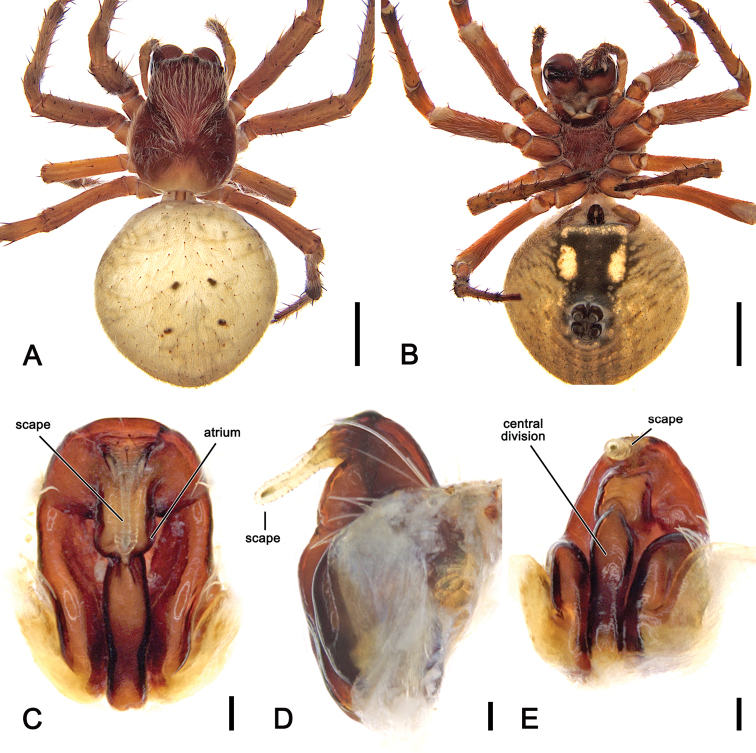
*Salsarueda* sp. nov., female (AM KS.50201) **A** dorsal habitus **B** ventral habitus **C** epigyne, ventral view **D** epigyne, lateral view **E** epigyne, posterior view. Scale bars: 2 mm (**A, B**); 0.1 mm (**C–E**).

**Figure 21.**
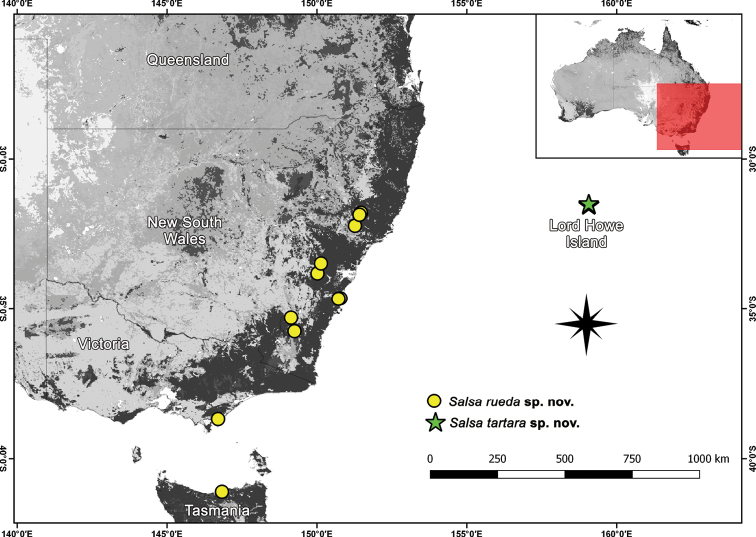
Distribution records of *Salsarueda* sp. nov. and *Salsatartara* sp. nov.

Figs 1B , 3A–D , 5E , 19A–D , 20A–E , 21

##### Type specimen.

***Holotype*** male, Tubrabucca (31°52'S, 151°25'E, New South Wales, Australia), 19 January 1049, RTMP, ANB (MV K-14856).

##### Other material examined.

6 males, 14 females (1 with egg sac), 1 juvenile (in 15 records) (see Suppl. material 1).

##### Etymology.

The specific epithet is a noun in apposition and refers to a specific Salsa dancing style, Rueda de Casino, in which changing pairs of dancers from a circle and dance moves are being called out by a single person. It is a noun in apposition.

##### Diagnosis.

Males of *S.rueda* sp. nov. are identified from all other species of the genus by the highly elongated median apophysis of the pedipalp and the enlarged basal, curved prong on the terminal apophysis (Figs 3A–C, 19C). Females can be distinguished from all other species by shape of the epigyne base, which is much longer than wide and has a central longitudinal ridge (Fig. 20A).

##### Description.

**Male** (based on holotype, MV K-14856). Total length 6.1. Carapace 3.3 long, 2.6 wide, brown, slightly paler in cephalic area and posteriorly (Fig. 19A). Eye diameter AME 0.16, ALE 0.14, PME 0.09, PLE 0.09; row of eyes: AME 0.47, PME 0.43, PLE 1.37. Chelicerae orange-brown; with four promarginal teeth (basal and apical largest) and three retromarginal teeth (basal largest). Legs shades of brown, femora basally yellow-brown in legs II, III and IV (Fig. 19A, B). Leg formula I > II > IV > III; length of segments (femur + patella + tibia + metatarsus + tarsus = total length): I – 4.5 + 1.5 + 2.8 + 2.6 + 1.0 = 12.4, II – 3.0 + 1.4 + 2.0 + 2.4 + 0.9 = 9.7, III – 1.7 + 0.9 + 1.2 + 1.2 + 0.6 = 5.6, IV – 2.6 + 1.1 + 1.8 + 2.2 + 0.8 = 8.5. Labium 0.36 long, 0.56, brown; endites brown (Fig. 19B). Sternum 1.5 long, 1.2 wide, dark brown (Fig. 19B). Abdomen 2.7 long, 2.6 wide, dorsal folium uniformly olive-grey bordered by broad wavy pale bands (Fig. 19A); venter dark olive-grey with two ovoid lateral white patches (Fig. 19B). Pedipalp length of segments (femur + patella + tibia + cymbium = total length): 0.6 + 0.2 + 0.1 + 1.1 = 2.0; paracymbium slightly curved with conspicuous base (Figs 3A, B, 19D); median apophysis bearing a rounded basal process, elongated C-shaped; basal arch with numerous tubercles (Figs 3A–D, 19C); conductor lobe broad (Figs 3A–C, 19C); terminal apophysis sub-rectangular with a curved, heavily sclerotised basal prong (Figs 3A–C, 19C); conductor heavily sclerotised, spatulate (Figs 3A–C, 19C); embolus strong and slightly sinuous (Figs 3A–C, 19C).

**Female** (based on AM KS.50201): Total length 10.5. Carapace 4.2 long, 3.5 wide; reddish brown, slightly paler in cephalic area and posteriorly, covered by white setae specifically in cephalic area (Fig. 20A). Eye diameter AME 0.18, ALE 0.16, PME 0.13, PLE 0.11; row of eyes: AME 0.54, PME 0.52, PLE 2.18. Chelicerae reddish brown, four promarginal teeth (apical and second basal largest) and three retromarginal teeth (basal largest). Legs orange-brown (Fig. 20A, B). Pedipalp length of segments (femur + patella + tibia + tarsus = total length): 1.1 + 0.4 + 0.7 + 1.3 = 3.5. Leg formula I > II > IV > III; length of segments (femur + patella + tibia + metatarsus + tarsus = total length): I – 4.0 + 1.7 + 3.5 + 3.3 + 1.2 = 13.7, II – 3.7 + 1.6 + 2.8 + 2.9 + 1.1 = 12.1, III – 2.5 + 1.1 + 1.4 + 1.5 + 0.8 = 7.3, IV – 3.5 + 1.6 + 2.2 + 2.6 + 1.0 = 10.9. Labium 0.58 long, 0.86 wide, dark brown; endites dark brown (Fig. 20B). Sternum 1.8 long, 1.6 wide, dark reddish brown (Fig. 20B). Abdomen 6.0 long, 6.0 wide; dorsum beige with indistinct darker folium pattern (Fig. 20A); venter black and laterally with elongate white patches and pale transverse band behind epigastric furrow (Fig. 20B). Epigyne much longer than wide; atrium with central elevated section and a transverse ridge anteriorly (Fig. 20C); scape shorter than half the length of epigyne base (Fig. 20C, D); central division a conspicuous narrow ridge (Fig. 20E). Spermathecae rounded and located on the basis of the genitalia, separated by the width of the median ridge (Fig. 5E).

##### Variation.

Total length males 6.0–6.8 (*n* = 5); females 7.2–10.5 (*n* = 4). The colour variations in *S.rueda* sp. nov. are probably the most uniform with the patterns in the folium often little expressed (Figs 19A, 20A). There was no evidence of scape breakage in any of the females examined by us.

##### Life history and habitat preferences.

Specimens were collected in December and January, with a single female from March, indicating this species to be summer-mature. There was no habitat information on any of the specimen labels.

##### Distribution.

*Salsarueda* sp. nov. were found in the Australian Capital Territory, New South Wales, Victoria, and Tasmania (Fig. 21).

#### ﻿ Salsa tartara sp. nov.

Taxon classificationAnimaliaAraneaeAraneidae

65712485-791F-5CA1-B70A-0A47610A58F0

http://zoobank.org/EADD2CE5-3A7B-4832-9D09-770F2BEA5ECB

**Figure 22.**
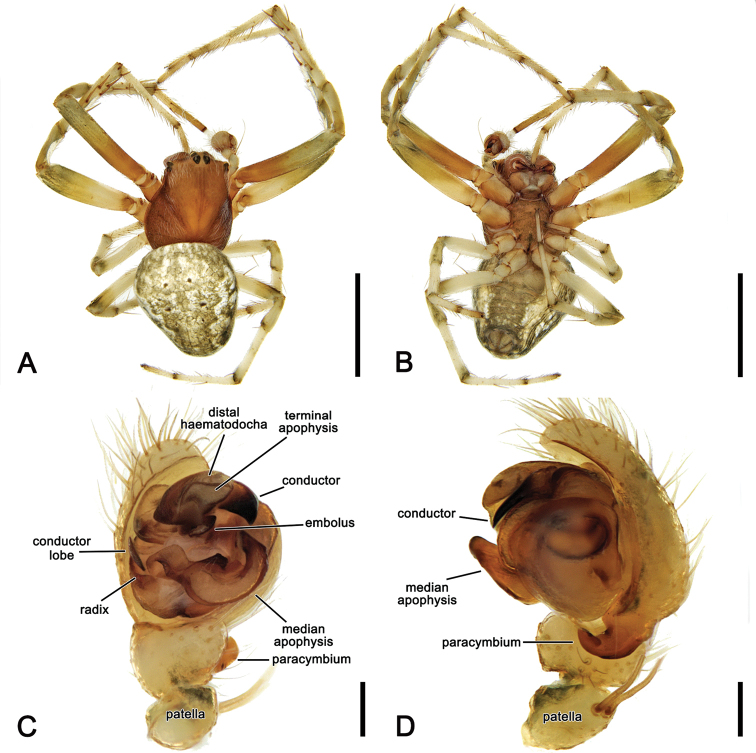
*Salsatartara* sp. nov., male holotype (AM KS.70737) **A** dorsal habitus **B** ventral habitus **C** left pedipalp, ventral view **D** left pedipalp, dorsal view. Scale bars: 2 mm (**A, B**); 0.2 mm (**C, D**).

**Figure 23.**
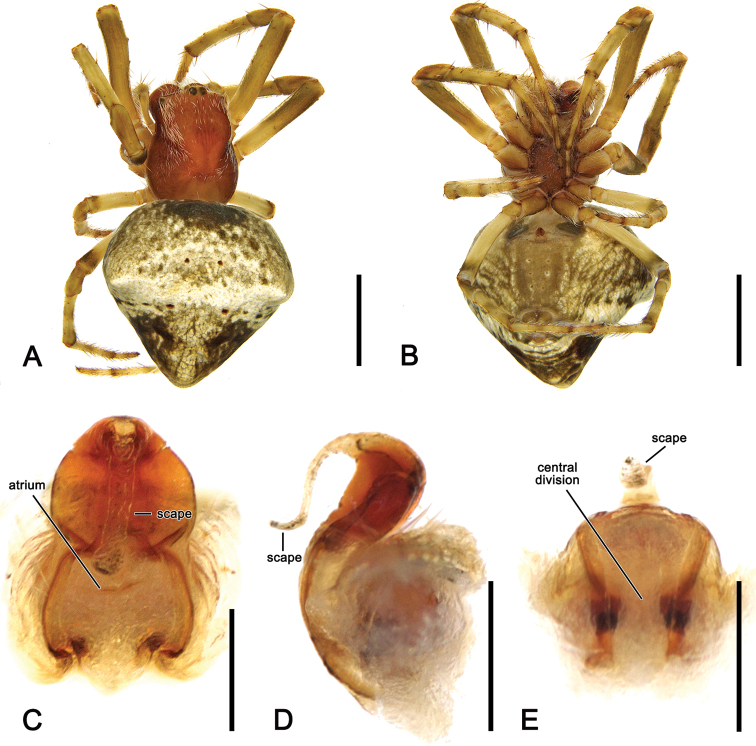
*Salsatartara* sp. nov., female (AM KS.70661) **A** dorsal habitus **B** ventral habitus **C** epigyne, ventral view **D** epigyne, lateral view **E** epigyne, posterior view. Scale bars: 2 mm (**A, B**); 0.2 mm (**C–E**).

Figs 5F , 21 , 22A–D , 23A–E

##### Type specimen.

***Holotype*** male, Lord Howe Island, Goat House Cave area (31°33'50"S, 159°05'11"E, New South Wales, Australia), 23 February 2001, G. Milledge (AM KS.70737).

##### Other material examined.

1 male, 5 females (in 6 records) (see Suppl. material 1).

##### Etymology.

The specific epithet is a noun in apposition and refers to the tartar sauce, “salsa tartara” in Spanish, one of the favourite salsas of the junior author’s wife.

##### Diagnosis.

Like *S.canalae* comb. nov. males, those of S. *tartara* sp. nov. have two patellar setae on the pedipalp; however, can be separated by the strong curved conductor (Fig. 22C) that is absent in *S.canalae* comb. nov. Female epigynes are much longer than wide, similar to those of *S.rueda* sp. nov., but they lack the longitudinal central ridge of that species (Fig. 20C vs. Fig. 23C).

##### Description.

**Male** (based on holotype, AM KS.70737) Total length 4.0. Carapace 2.1 long, 1.7 wide, brown, slightly paler in cephalic area (Fig. 22A). Eye diameter AME 0.14, ALE 0.13, PME 0.09, PLE 0.09; row of eyes: AME 0.43, PME 0.38, PLE 1.40. Chelicerae orange-brown; with four promarginal teeth (second basal largest) and three retromarginal teeth (basal largest). Legs yellowish brown mottled in grey on joints; femora I and II basally orange-brown (Fig. 22A, B). Leg formula I > II > IV > III; length of segments (femur + patella + tibia + metatarsus + tarsus = total length): I – 2.4 + 1.1 + 1.9 + 1.9 + 0.8 = 8.1, II – 2.0 + 0.9 + 1.6 + 1.7 + 0.7 = 6.9, III – 1.2 + 0.6 + 0.7 + 0.7 + 0.45 = 3.65, IV – 1.6 + 0.7 + 1.2 + 1.2 + 0.6 = 5.3. Labium 0.31 long, 0.45, brown; endites orange-brown (Fig. 22B). Sternum 1.0 long, 0.8 wide, orange-brown with dusky discolourations (Fig. 22B). Abdomen 2.2 long, 1.9 wide, dorsum with beige background and olive-grey, irregular folium, laterally dark olive-grey with dark streaks (Fig. 22A); venter olive-brown, laterally with thin, irregular white lines (Fig. 22B). Pedipalp length of segments (femur + patella + tibia + cymbium = total length): 0.4 + 0.15 + 0.15 + 0.6 = 1.3; paracymbium short with pronounced base and slightly curved apically (Fig. 22D); median apophysis C-shaped, basally pronounced and with an acute and apically curved pointed tip (Fig. 22C); conductor lobe spatulate (Fig. 22C); terminal apophysis sub-rectangular; conductor strongly sclerotised and curved basally (Fig. 22C); embolus short and strongly sclerotised.

**Female** (based on AM KS.70661): Total length 6.5. Carapace 2.5 long, 2.1 wide; colouration and setae largely as in male (Fig. 23A). Eye diameter AME 0.16, ALE 0.14, PME 0.11, PLE 0.10; row of eyes: AME 0.47, PME 0.45, PLE 1.92. Chelicerae colour hue as in male, four promarginal teeth (apical and second basal largest) and three retromarginal (basal largest). Legs similar to male but leg I femora basally not orange (Fig. 23A, B). Pedipalp length of segments (femur + patella + tibia + tarsus = total length): 0.7 + 0.3 + 0.4 + 0.8 = 2.2. Leg formula I > II > IV > III; length of segments (femur + patella + tibia + metatarsus + tarsus = total length): I – 2.4 + 1.1 + 1.9 + 2.0 + 0.8 = 8.2, II – 2.0 + 1.0 + 1.7 + 1.6 + 0.7 = 7.0, III – 1.4 + 0.6 + 0.7 + 0.7 + 0.5 = 3.9, IV – 1.9 + 0.9 + 1.3 + 1.4 + 0.7 = 6.2. Labium 0.18 long, 0.29 wide, brown; endites dark brown (Fig. 23B). Sternum 1.2 long, 1.0 wide, dark brown (Fig. 23B). Abdomen 4.0 long, 4.2 wide, sub-triangular with distinct humeral humps, dorsally mottled olive-grey and white, with darker spots anteriorly and postero-laterally; folium pattern indistinct (Fig. 23A); venter as in male (Fig. 23B). Epigyne plate longer than wide and composed of two separate sections; borders thin and atrium wide; scape slightly longer than half of epigyne base, sinuous in lateral view (Fig. 23C, D); central division almost sub-rectangular, somewhat wider ventrally (Fig. 23E). Spermathecae enlarged, occupying most of the epigyne area (Fig. 5F).

##### Variation.

Only one additional male was measured, total length 5.1; females 5.2–7.2 (*n* = 5). Four of the five specimens we analysed had broken-off scapes. There is little colour variation in the specimens examined for this study, although most females have a more prominent folium, similar to the male examined here.

##### Life history and habitat preferences.

All mature specimens of *S.tartara* sp. nov. were collected in February and March, but collection numbers are too low to interpret the phenology of this species and may reflect a collection bias of expeditions to Lord Howe Island. But it appears that the species is (late) summer-mature to autumn-mature. There is no habitat information on the labels of any of the specimens collected, with the exception of one specimen collected in ‘litter’.

##### Distribution.

*Salsatartara* sp. nov. is currently only known from Lord Howe Island and should be considered endemic to this island (Fig. 21).

## ﻿Discussion

Recent large-scale molecular studies of world-wide Araneidae (e.g., Kallal and Hormiga 2018; Kallal et al. 2018; Scharff et al. 2020) have transformed our understanding of the evolutionary history of the family, in particular as it applies to the Australian fauna. The subfamily Araneinae Clerck, 1757 as circumscribed by Scharff and Coddington (1997) based on a preliminary morphological phylogenetic analysis has been shown to be highly paraphyletic and Australian taxa fall into a number of new groupings at the subfamily level, such as ‘backobourkiines’ and ‘zealaraneines’ (Scharff et al. 2020). These groupings, although well supported statistically, were not assigned formal subfamily status due to their limited taxonomic and systematic knowledge. However, they now allow us to tackle the taxonomy of Australian araneids in a much more systematic fashion, including our ongoing extensive revision of the ‘backobourkiines’ of which the current study forms a part (e.g., Framenau et al. 2010, 2021a, c, 2022; Framenau 2011). Male genitalic characters that unite the backobourkiines include a basal arch of the median apophysis that reaches over the radix and the presence of a single patellar spine (i.e., two patellar spines in eriophorines and zealaraneines) (Scharff et al. 2020).

In *Salsa* gen. nov. the arch of the median apophysis is internally armed with numerous small denticles (e.g., Fig. 3A, 9C). Modifications of this arch in other genera are not uncommon. In *Backobourkia*, the basal arch is apically extended into a long flange (Framenau et al. 2010). In an undescribed genus represented by *Araneusdimidiatus* (L. Koch, 1871) and *Araneusmulierarius* (Keyserling, 1887) (“NGEN03” in Scharff et al. 2020) there is a single long spine inside the arch (VWF unpublished data). The latter was not part of the backobourkiines in Scharff et al.’s (2020) study but formed a statistically unsupported clade with the largely Australian *Dolophones* Walckenaer, 1837 and the cosmopolitan *Cyclosa* Clerck, 1757. However, there is good morphological support of NGEN03 to be part of the backobourkiines as they have the two putative synapomorphies of the male pedipalp as mentioned above (VWF unpublished data). The functional role of these basal modifications of the median apophysis are not known, but it is perceivable that internal tubercles or a spine play a role in stabilising the link between the median apophysis and the radix during the expansion of the pedipalp during copulation.

Two species of *Salsa* gen. nov., namely *S.canalae* comb. nov. and *S.tartara* sp. nov., have two spines on the male pedipalp patella. Two patellar spines appear more common in traditional araneine genera (see Scharff and Coddington 1997) and are also present in eriophorines and zealaraneines as defined by Scharff et al. (2020). It therefore appears that the presence of two spines may represent the plesiomorphic condition and therefore a reversal in those two *Salsa* gen. nov. species amongst the backobourkiines with only a single spine. This reversal to two patellar spines has similarly occurred in *Hortophoracucullus* Framenau & Castanheira, 2021 (Framenau et al. 2021a), but the evolutionary significance, i.e., the functional roles of these spines, remains unknown.

*Salsa* gen. nov. clearly constitutes a natural grouping within the backobourkiines and is well diagnosed by genitalic and somatic characters, such as the C-shaped median apophysis of the male pedipalp, the single posterior abdominal hump or the ventral colouration of the abdomen. Molecular data places *Salsa* gen. nov. in a clade with *Acroaspis* and *Socca* (Scharff et al. 2020) and this association is supported by characters of the male pedipalp, in particular the shape of the sclerite that we considered the terminal apophysis. It is a sclerite, that amongst the Araneidae as a whole is difficult to homologise. It originates apically at the embolic division together with the embolus and, if present, the subterminal apophysis. These structures arise from the stipes (see Coddington 1990; Comstock 1910), the latter sclerite being poorly defined in *Salsa* gen. nov., if present at all. In the backobourkiines, we can identify two major shapes of the terminal apophysis: in *Backobourkia*, *Lariniophora*, *Novakiella*, and *Hortophora* it is inflated and sometimes bubble-shaped with a terminal spine, and in *Plebs*, *Socca*, *Acroaspis*, and *Salsa* gen. nov. it is flat lamellar, sometimes with processes (Framenau et al. 2010, 2021a, c, 2022; Framenau 2011, 2019; Joseph and Framenau 2012;). The terminal apophysis of *Salsa* gen. nov. is most similar to the one of *Socca* and *Acroaspis*, with a basal shape of a triangular to sub-rectangular plate (Fig. 6C, 9C, 17C). In *Acroaspis*, this plate is covered centrally by an elongate, triangular and lamellar process, at least in the only species with a published illustration, *Acroaspislancearia* (Keyserling, 1887) (Framenau 2019: fig. 1B). In *Socca* this structure is further modified so that the lamellar process divides the terminal apophysis plate to form a tri-partite complex (Framenau et al. 2022).

The epigynes of most *Salsa* gen. nov. have a large exposed plate, except for *S.fuliginata* comb. nov. (Fig. 7F). However, a comparison with *S.recherchensis* comb. nov. shows an intriguing ‘twist’. Both epigynes are in fact very similar, but its base in *S.fuliginata* comb. nov. is rotated into the abdomen, illustrating that the boundary between the atrium and central division is somewhat arbitrary and depending on the position of the epigyne. The posterior view in *S.fuliginata* comb. nov. and the ventral view in *S.recherchensis* comb. nov. views are very similar between the two species displaying a heart-shaped atrium/central division (Fig. 7C, E vs. Fig. 18C, F). This epigyne rotation is not present in any other backobourkiine we have treated so far, and is not known to us in any other araneid genera, and suggests caution when trying to homologise structures in the epigyne based on position.

*Salsa* gen. nov. is a largely Australian genus, but contains three ‘island’ endemics, which are, based on our current knowledge, only present on Lord Howe Island, New Caledonia, and Papua New Guinea. A single species was introduced from Australia to New Zealand, but the means of this introduction, i.e., natural or facilitated by man, are unknown. Similar distribution patterns can be found in other backobourkiines, all of which have the centre of their distribution in Australia. The most widespread genus is *Plebs*, species of which can be found from Australia into SE Asia, China, and India (Joseph and Framenau 2012). *Hortophora* is also mainly Australian, but some species are found in the Pacific region (Framenau et al. 2021a). *Backobourkia* and *Novakiella* are exclusively Australian, although just like in *Salsa* gen. nov., one species each was introduced to New Zealand (Framenau et al. 2010, 2021c). The same seems the case for *Acroaspis*, but until the genus is taxonomically revised in detail, it remains unclear if the single New Zealand species, *A.decorosa* (Urquhart, 1894) can also be found in Australia. The distribution of *Carepalxis* currently includes the Nearctic but a recent study suggests that the first males described from there are not conspecific with the Australian species (Ferreira-Sousa and Motta 2022). The type species of *Carepalxis*, *C.montifera* L. Koch, 1872, is from Australia, but as in *Acroaspis*, further biogeographic analyses require a detailed revision of the genus.

The presence of *S.fuliginata* comb. nov. in New Zealand is curious. First records of the species in the country date back to the late 1800s, as two females of the Graf Erich von Keyserling (1833–1889) collection are present in the NHMUK (see Material examined of that species). However, the species was not included in a comprehensive revision of New Zealand’s large orb-weaving spiders (Court and Forster 1988) and it must be assumed that the species did not persist in the country following the records from the late 1800s. Recent records based on museum specimens and images support the presence of the species only from 2008 with a female imaged in Hamilton on the North Island by B. McQuillan (Fig. 1C). However, it is also possible that the historic females in the NHMUK collection were mislabeled specimens from Australia.

## Supplementary Material

XML Treatment for Salsa

XML Treatment for Salsa fuliginata

XML Treatment for Salsa brisbanae

XML Treatment for Salsa canalae

XML Treatment for Salsa neneba

XML Treatment for Salsa recherchensis

XML Treatment for Salsa rueda

XML Treatment for Salsa tartara

## Supplementary materials

Supplementary material 1 Other material examined Data type docx file Explanation note Other material examined: *Salsafuliginata* comb. nov., *Salsabrisbanae* comb. nov., *Salsacanalae* comb. nov., *Salsarecherchensis* comb. nov., *Salsarueda* sp. nov., *Salsatartara* sp. nov. https://binary.pensoft.net/file/686623This dataset is made available under the Open Database License (http://opendatacommons.org/licenses/odbl/1.0/). The Open Database License (ODbL) is a license agreement intended to allow users to freely share, modify, and use this Dataset while maintaining this same freedom for others, provided that the original source and author(s) are credited.Volker W. Framenau, Pedro de S. Castanheira
