# Supplementary material for: ﻿Revision of the new Australasian orb-weaving spider genus Salsa (Araneae, Araneidae)
Source: Zookeys. 2022 May 20;1102:107–48. doi: 10.3897/zookeys.1102.82388 (PMC9848856; doi:10.3897/zookeys.1102.82388)
Supplement: Supplementary material 1 — Other material examined [file zookeys-1102-107_article-82388__-s001.docx]

**Supplementary file**

**Other material examined**

***Salsa fuliginata* comb. nov.**

AUSTRALIA – **Australian Capital Territory •** 2 females, 1 juv.; Canberra; 35º18'S, 149º08'E; AM KS.28873 **•** 4 females; same collection data as preceding; QM **•** 1 female; same collection data as preceding; QM S41081 **•** 1 male, 2 females; Canberra; Najor Orchard; 35º18'S, 149º08'E; AM KS.33141 **•** 10 females; same collection data as preceding; QM **•** 1 male; Cook; Grylls Crescent; 35º16'S, 149º04'E; WAM T74445 **•** 1 female; Lees Spring; 35º20'S, 148º53'E; MV K-9374; **New South Wales •** 1 female; Agnes Banks; 33º37'S, 150º41'E; AM KS.33337 **•** 1 female; ‘Andrewville’; Kangaloon Road; Glen Quarry; 34º34'S, 150º35'E; AM KS.67211 **•** 1 female, 2 juv.; Antonio Creek; 33º32'S, 150º01'E; AM KS.34005 **•** 1 male; same collection data as preceding; AM KS.68779 **•** 11 females, 2 juv.; Barrington Tops; 31º58'S, 51º26'E; AM KS.33381 **•** 2 females; same collection data as preceding; AM KS.32696 • 1 female; same collection data as preceding; AM KS.48705 • 1 female; same collection data as preceding; MV K9445 • 1 male; Beecroft; 33º45'S, 151º04'E; AM KS.51879 • 1 female; Beecroft; Observatory Park; 33º45'S, 151º04'E; AM KS.71742 • 1 female; Bilpin; 33º30'S, 150º31'E; AM KS.32642 • 2 males, 2 females, 7 juv., Blackheath; 33º38'S, 150º17'E; MV K9364 • 1 female; Blue Mountains; 33º30'S, 150º23'E; AM KS.65922 • 1 female; Bowral; 10km SE; 34º32'50''S, 150º29'20''E; AM KS.51911 **•** 1 female; same collection data as preceding; AM KS.51916 **•** 1 female; same collection data as preceding; AM KS.51918 **•** 1 female; same collection data as preceding; AM KS.51917 **•** 1 male; same collection data as preceding; AM KS.51914 • 1 female; Bungonia; 34º48'S, 150º00'E; AM KS.22747 • 1 female; Carrow Brook; 32º17'S, 151º18'E; AM KS.58623); 1 female; same collection data as preceding; AM KS.58627 • 1 female; Colo Vale; 34º24'S, 150º29'E; AM KS.32945 • 1 male; Coolah Tops National Park; Cox's Creek Campground; 31º44'00''S, 150º00'30''E; AM KS.75055 • 1 male; Coolah Tops National Park; Grass Tree Track; 31º44'06''S, 150º00'05''E; AM KS.75114 • 1 female; Coonabarabran; ‘Smoky Hollow’; 31º16'S, 149º17'E; AM KS.7544 • 1 female, 4 juv.; Daners Gap, Mt Kosciusko; 36º22'S, 148º29'E; AM KS.34030 • 1 female; Dawsons Spring; Mt Kaputar; 30º16'30''S, 150º9'E; QM • 1 female; Dobroyd Head; Sydney; 33º48'S, 151º17'E; WAM T73575 • 1 female; Enfield; 33º53'S, 151º06'E; AM KS.32861 • 1 female, 2 juv.; same collection data as preceding; AM KS.32864); 1 female; same collection data as preceding; AM KS.32863 • 1 male; Euroka; 33º45'S, 150º13'E; AM KS.32895 • 1 female; Frying Pan; Cooma; 36º09'S, 148º50'E; AM KS.54056 • 1 female; same collection data as preceding; AM KS.54061 • 1 male; same collection data as preceding; AM KS.58685 • 1 female; same collection data as preceding; AM KS.54067 • 1 male; same collection data as preceding; AM KS.54064 • 1 female; same collection data as preceding; AM KS.54449 • 1 male; same collection data as preceding; AM KS.54066 • 1 male; same collection data as preceding; AM KS.54068 • 1 female; same collection data as preceding; AM KS.54069 • 1 female; same collection data as preceding; AM KS.53764 • 1 female; same collection data as preceding; AM KS.58679 • 1 female; same collection data as preceding; AM KS.51568 • 1 female; Goat Island, River Murray; 34º16'S, 140º32'E; AM KS.34027 • 1 female; Gosford; 33º25'S, 151º20'E; MV K9724 • 1 female; Green Point; 33º28'S, 151º21'E; AM KS.50832 • 1 female; Guthega; 36º21'S, 148º25'E; AM KS.45448 • 1 male; Hunter Valley; 32º44'S, 151º34'E; AM KS.7305 • 1 male; Jamberoo Mountain; 34º40'S, 150º43'E; AM KS.49936 • 1 male; same collection data as preceding; AM KS.57123 • 1 male; same collection data as preceding; AM KS.54105 • 1 male; same collection data as preceding; AM KS.49932); 1 female; same collection data as preceding; AM KS.49963 • 1 female; same collection data as preceding; AM KS.66244 • 1 male; same collection data as preceding; AM KS.49935 • 1 female; same collection data as preceding; AM KS.58625 • 1 female; Jenolan Caves; 33º49'18''S, 150º01'20''E; AM KS.30291 • 13 females; same collection data as preceding; AM KS.32693 • 1 female; Katoomba District; 33º43'S, 150º19'E; AM KS.7865 • 4 females; Kosciusko; 36º27'S, 148º16'E; AM KS.32686 • 1 female; Lachlan River; Whealbar Bridge; 34º05'S, 149º01'E; MV K9421 • 1 female; Lachlan River; Whealbar Bridge; 34º05'S, 149º01'E; MV K9420 • 1 male; Lemann Property; ca. 10 km SE; 34º37'50''S, 150º29'30''E; AM KS.54223 • 1 female; Leura; 33º43'S, 150º19'E; AM KS.33318 • 4 females, 1 juv.; same collection data as preceding; AM KS.33732 • 16 females, 4 juv.; Lithgow; 33º29'S, 150º08'E; AM KS.32865 • 1 male; 1 female; same collection data as preceding; AM KS.32689 • 1 male; Livingstone National Park; 35º37'06''S, 147º35'31''E; AM KS.92640 • 1 male; same collection data as preceding; AM KS.92625 • 5 females; Llangothlin; 30º08'S, 151º41'E; AM KS.34138 • 19 females, 5 juv.; same collection data as preceding; AM KS.33545 • 2 females; Manning River; 31º55'S, 151º28'E; AM KS.33371 • 16 females, 1 juv.; Medlow Bath; 33º41'S, 150º17'E; AM KS.33544 • 4 females; Minnamurra Falls; 34º38'S, 150º44'E; AM • 1 female; Mittagong; 34º27'S, 150º27'E; AM KS.34093 • 1 female; Montague Island; 36º15'S, 150º14'E; AM KS.19928 • 2 females; Mosman; 33º50'S, 151º15'E; AM KS.3262 • 2 females, 2 juv.; Mosman; same collection data as preceding; AM KS.34125 • 2 males; Mt Nelson; behind Hytten Hall; 42º56'S, 147º20'E; AM KS.51910 • 1 female; Mt Piddington; near Mt Victoria; 33º37'S, 150º15'E; AM KS.46072 • 1 female; Mt Victoria; 33º35'S, 150º15'E; AM KS.34363 • 1 male, 1 female; New England National Park; 30º29'S, 152º30'E; QM • 1 female; Newnes State Forest; Birds Rock Flora Reserve; 0.6km from Sunnyside Ridge Road; 33º19'43''S, 150º11'33''E; AM KS.94506 • 1 male; Newnes State Forest; Blackfellows Hand Road; 0.5km from Sunnyside Road; 33º23'11''S, 150º10'44''E; AM KS.93148 • 5 males; Newnes State Forest; Blackfellows Hand Road; 5.9km from Sunnyside Road; 33º21'26''S, 150º08'40''E; AM KS.92985 • 1 male; Newnes State Forest; Sunnyside Road; 1.6km from Blackfellows Hand Road; 33º22'33''S, 150º11'14''E; AM KS.93216 • 1 male; same collection data as preceding; AM KS.93192 • 1 male; same collection data as preceding; AM KS.93190 • 1 male; same collection data as preceding; AM KS.93189 • 1 female; North Ryde; 33º49'S, 151º06'E; AM KS.34153 • 1 female; Polblue; 31º57'S, 151º27'E; MV K9801 • 3 males, 4 females, Pomingalarnaa Park; 8km W of Wagga Wagga; 35º04'S, 147º22'E; AM KS.93984 • 2 males, 3 females; same collection data as preceding; AM KS.94015 • 1 female, 1 juv.; same collection data as preceding; AM KS.93827 • 4 females; same collection data as preceding; AM KS.94022 • 4 males; same collection data as preceding; AM • 1 male, 4 females; same collection data as preceding; AM KS.93883 • 1 male; same collection data as preceding; AM KS.93994 • 1 male; same collection data as preceding; AM KS.93874 • 1 female; Pullitop; via Wagga; 35º07'S, 147º22'E; AM KS.34071 • 1 female; Snowy Mountains; Junction Shaft; no exact locality; QM • 1 female; Stephens Creek; 16km N Broken Hill; 31º49'S, 141º31'E; MV K9785 • 1 female, 1 juv.; Sydney; 33º53'S, 151º13'E; ZMB • 1 female; Thredbo; 36º30'S, 148º19'E; AM KS.58711 • 1 female; same collection data as preceding; AM KS.53766 • 1 female; same collection data as preceding; AM KS.53767 • 1 female; same collection data as preceding; AM KS.54101 • 1 female; same collection data as preceding; AM KS.53765 • 1 female; same collection data as preceding; AM KS.54461 • 1 female; same collection data as preceding; AM KS.54072 • 1 female; same collection data as preceding; AM KS.54465 • 1 female; same collection data as preceding; AM KS.54071 • 1 female; same collection data as preceding; AM KS.54468 • 1 female; same collection data as preceding; AM KS.54463 • 1 female; same collection data as preceding; AM KS.54467 • 1 female; same collection data as preceding; AM KS.54466 • 1 female; Tinderry Range; 17.8km from Captains Flat Road jct, c. 2km W of Calabash Road on Tinderry Road; 35º45'01''S, 149º15'40''E; WAM T68094 • 1 male, 1 female, 3 juv.; Tomalla; 31º48'S, 151º28'E; MV K9769 • 1 female; Toolybue, no exact locality; SAM • 1 female; Toowoon Bay; 33º22'S, 151º29'E; AM KS.34702 • 2 males, 3 females; Tubrabucca; 31º52'S, 151º25'E; MV K9478 • 1 female; same collection data as preceding; MV K9359 • 2 females; same collection data as preceding; MV K9366 • 2 females, 1 juv.; same collection data as preceding; MV K9463 • 4 males; same collection data as preceding; MV K9846 • 7 males, 7 females; same collection data as preceding; MV K9475 • 1 female; same collection data as preceding; MV K9404 • 2 females; same collection data as preceding; MV K9367 • 7 males, 4 females; same collection data as preceding; MV K9723 • 1 male; same collection data as preceding; MV K9758 • 1 male; same collection data as preceding; MV K9842 • 2 males, 2 females, 4 juv.; same collection data as preceding; MV K9362 • 1 female; Tubrabucca; Upper Hunter District; 31º58'S, 151º28'E; AM KS.65008 • 1 female; Tubraburra; 31º53'S, 151º25'E; AM KS.33731 • 3 females, 2 juv.; Upper Tumut Gorge; Snowy Mountains; 35º29'S, 148º21'E; QM • 1 male; Wambo Colliery at Warkworth Sands; Wollombi Brook; 32º03'05''S, 151º00'22''E; AM KS.92220 • 1 male; Wamboin; 6 Cooper Road; 35º15'33''S, 149º16'46''E; WAM T67910 • 1 female; Washpool State Forest; swamp area on Gwider Highway, 29º16'S, 152º21'E; AM KS.9091 • 1 female; same collection data as preceding; AM KS.9219 • 2 females; Woodford; 33º44'S, 150º29'E; AM KS.32884 • 3 females, 17 juv.; Woodford; 33º44'S, 150º29'E; AM KS.32858 • 5 females; Yanco; 34º36'S, 146º25'E; AM KS.33333 • 1 female; Yaredimbah; 33º28'S, 144º59'E; AM KS.32680 • 1 female; Yarramundi; 33º37'S, 150º40'E; AM KS.34044 • 1 male; same collection data as preceding; AM KS.34106 • 1 female; Yass; 34º51'S, 148º55'E; AM KS.33400; **South Australia** • 1 male; Aldgate; 35º00'S, 138º44'E; SAM • 1 female; Balah Station; 33º46'S, 139º51'E; SAM • 1 female; Ewens Ponds; 38º02'S, 140º48'E; SAM NN24281 • 1 female; Fullarton; 34º57'S, 138º37'E; NHMD • 1 female; Mt Gambier; 37º49'S, 140º46'E; SAM NN24282 • 1 male; Parawa; 2km WNW; 35º33'16''S, 138º20'05''E; SAM • 1 female; Penola Forest Reserve, Topperweins Scrub; 37º28'S, 140º51'E; SAM NN24283 • 2 females; Robe; 37º9'S, 139º45'E; SAM • 1 male, 1 female; Sellicks-Aldinga Scrub; 35º17'S, 138º27'E; SAM NN24284-5 • 1 female; Whyalla; 33º02'S, 137º34'E; MV K-9771; **Tasmania** • 1 male; Babel Island; Furneaux Group; 39º56'S, 148º19'E; MV K9834 • 1 male, 1 female, 1 juv.; Babel Island; Furneaux Group; 39º56'S, 148º19'E; MV K9365 • 1 male; same collection data as preceding; MV K9847 • 1 female; Beehive Canal; 42º11'S, 146º05'E; QM • 1 female; Birthday Bay and Hibbs Lagoon, between; 42º30'S, 145º16'E; QM • 2 males, 1 female; Bracknell; 41º39'S, 146º56'E; QVMAG 13:0056 • 1 female; Bridgeport; 41º00'S, 147º23'E; AM KS.65758 • 2 females; Burns Dam; 42º11'S, 146º06'E; QM S72276 • 1 female; Coates Creek; 42º10'S, 146º10'E; QM • 1 female; Condominium Creek; 42º57'S, 146º22'E; QM S80898 • 1 female; Currawong; 33º36'S, 151º18'E; AM KS.34079 • 1 female; Derwent Bridge; Road between Qtown & L. Claire; 42º08'S, 146º13'E; NHMD • 2 males, 2 females, Domain; 42º52'S, 147º19'E; AM KS.28827 • 1 female; Edgar; 43º00'S, 146º20'E; QM S77751 • 3 males, 1 female; Franklin River; 42º27'25''S, 145º43'45''E; QM • 1 female; Harbacks Road; 42º12'S, 146º11'E; QM S72283 • 1 female; King Island; 39º53'S, 143º54'E; MV K9393 • 3 females; same collection data as preceding; MV K9396 • 1 female; same collection data as preceding; MV K9467 • 1 female; same collection data as preceding; MV K9376 • 2 males; same collection data as preceding; MV K9850 • 2 females; King William Creek; 42º11'S, 146º08'E; QM • 1 female; same collection data as preceding; QM S72725 • 1 female; same collection data as preceding; QM • 2 females; same collection data as preceding; QM • 1 male; same collection data as preceding; QM • 1 female; same collection data as preceding; QM • 1 female; same collection data as preceding; QM • 1 female; same collection data as preceding; QM • 1 female; same collection data as preceding; QM • 1 female; Kingston; 42º59'S, 147º18'E; AM KS.32690 • 1 female; Lake St Clair; 42º03'S, 146º10'E; QM S5646 • 1 male, 4 juv.; same collection data as preceding; QM • 1 male; same collection data as preceding; QM • 1 male, 2 females; same collection data as preceding; QM S77907 • 3 males; same collection data as preceding; QM • 2 females; same collection data as preceding; QM S5582 • 1 female; same collection data as preceding; QM S77923 • 1 female, 3 juv.; same collection data as preceding; QM S5561 • 1 male; same collection data as preceding; QM S5585 • 2 males; same collection data as preceding; QM • 1 female; same collection data as preceding; QM S77906 • 1 female; same collection data as preceding; QM • 1 female; Lake St Clair Road; 42º03'S, 146º10'E; QM S72282 • 1 female; same collection data as preceding; QM S72279 • 2 females; same collection data as preceding; QM S72281 • 1 female; same collection data as preceding; QM S72280 • 3 females; Launceston; 41º27'S, 147º10'E; AM KS.28864 • 1 female; Launceston; same collection data as preceding; AM KS.28865 • 1 female; Launceston; 41º26'S, 147º08'E; TMAG J323 • 1 female; McPartlan Pass; 42º51'S, 146º11'E; QM • 1 female; same collection data as preceding; QM • 1 female; same collection data as preceding; QM S68050 • 1 female; same collection data as preceding; QM S70406 • 1 male; same collection data as preceding; QM S77972 • 1 male; Mt Wellington; 42º54'S, 147º14'E; AM KS.28632 • 1 male; 1 juv.; same collection data as preceding; QM S11603 • 3 males, 2 females, 1 juv.; same collection data as preceding; QM S11606 • 2 females; Navarre Plains; 42º10'S, 146º10'E; QM S80947 • 1 female; same collection data as preceding; QM • 1 female; same collection data as preceding; QM S72277 • 2 males, 1 female; New Town; 42º53'S, 147º19'E; AM KS.28849 • 1 male, 1 female; same collection data as preceding; AM KS.28638 • 4 males, 4 females; same collection data as preceding; AM KS.28700 • 2 males, 2 females; same collection data as preceding; AM KS.28640 • 1 female; North Lund Karst area; NI-9 (Top Sink); 43º24'S, 146º50'E; AM KS.48760 • 1 male, 2 females; Orford; 42º34'S, 147º52'E; AM • 1 female; Queenstown; 42º04'S, 145º33'E; MV K9387 • 1 female; Ridgeway; 42º55'S, 147º17'E; MV K9473 • 1 female; Risdon; 42º49'S, 147º21'E; AM KS.28838 • 1 female; Rufus Canal Road; 42º10'S, 146º07'E; QM • 1 female; Sassafras Creek; near Hole Creek; 41º35'S, 146º20'E; QM • 1 female; Still Water; 41º26'S, 147º08'E; QM S77766 • 1 female; same collection data as preceding; QM S77767 • 1 female; Strahan; 42º9'S, 145º19'E; AM KS.34087 • 2 females; Strathgordon Caravan Park; 42º45'S, 146º03'E; QM S`5747 • 1 male; SW Tasmania; 42º38'S, 145º55'E; AM KS.27431 • 4 females; Tarraleah; 42º18'S, 146º26'E; AM KS.28848 • 1 female; same collection data as preceding; AM • 1 female; Travellers Rest; 41º29'S, 147º05'E; QM S72274 • 2 females; Waddamana; 42º08'S, 146º45'E; AM KS.44690 • 1 male; Waldheim Forest; Cradle Mountain National Park; 41º38'S, 145º56'E; QM S5541 • 3 females; Weatherstation, no exact locality; QM S72273 • 1 female; WHA vial 20-19, no exact locality; QM; **Victoria** • 2 females, no exact locality; MV K-9392 • 2 females; no exact locality; MV K-9383; 2 females; no exact locality; MV K-9375 • 2 females; no exact locality; MV K-9388 • 10 females, 3 juv.; no exact locality; MV K-9434 • 1 female; no exact locality; MV K-9391 • 1 female, 1 juv.; Alexandra; 37º11'S, 145º42'E; MV K-9382 • 2 females; Altona Bay; 37º52'S, 144º49'E; MV K-9457 • 1 female; Bacchus Marsh; 37º40'S, 144º26'E; MV K-9381 • 1 female; 6.5 km NE Bacchus Marsh; Longforest Road; 37º35'S, 144º30'E; WAM T74438 • 1 male, 1 juv.; 2 km E Beaufort; 37º25'S, 143º23'E; SAM • 1 female; Bendigo; 36º46'48''S, 144º18'00''E; MV K-15858 (CVIC 1168) • 2 females; Berwick; Quarry Hill; 38º02'S, 145º20'E; QM • 1 female; Bruthen; 37º42'S, 147º50'E; MV K-9390 • 1 male; Buxton; 37º25'S, 145º42'E; MV K-9840 • 1 female; Camberwell; 37º50'S, 145º04'E; MV K-9414 • 1 female, 1 juv.; Carnegie; 37º53'S, 145º03'E; MV K-9389 • 1 female; Cockatoo; 37º56'S, 145º29'E; MV K-9405 • 1 male, 2 females; Coolah Valley; 31º50'S, 149º43'E; AM • 1 female, 1 juv.; Coranderrk Reserve; 37º41'S, 145º31'E; WAM T73576 • 1 female; Creswick; 37º25'S, 143º53'E; MV K-9407 • 2 females; Croydon; 37º47'S, 145º16'E; MV K-9386, K-9433 • 2 females; same collection data as preceding; MV K-9454 • 1 male; same collection data as preceding; MV K-9843 • 4 females; same collection data as preceding; MV K-9369 • 1 female, 1 juv.; Dartmoor; 37º55'S, 141º16'E; MV K9452 • 1 male, 1 juv., Delatite River; at Merrigig; 37º07'S, 146º12'E; QM • 1 male; Dondangadale; 36º47'S, 146º40'E; MV K-9845 • 1 female; Echuca; 36º08'S, 144º45'E; MV K-9379 • 1 female, 1 juv., Flowerdale; 37º19'S, 145º17'E; MV K-9377 • 1 female; Forrest Caravan Park; 38º31'S, 143º43'E; WAM T74446 • 2 females, 1 juv.; Foster; 38º39'S, 146º12'E; MV K-9739 • 10 females, 2 juv.; Gifford; 37º46'S, 145º49'E; MV K-9449 • 1 male; Glenisla; 37º13'S, 142º11'E; MV K-9852 • 1 female; Granite Mountain; 9 km NW Buldah; 37º05'S, 149º03'E; MV K-9417 • 3 females; Hamilton; 37º44'S, 142º01'E; MV K-9415 • 1 male, 1 female; Hamilton; 37º44'S, 142º01'E; MV K-9462 • 1 female; Hamilton; 37º44'S, 142º01'E; MV K-9774 • 2 males; Hanging Rock; 37º01'S, 148º19'E; SAM • 1 male; Heidelberg; 37º45'S, 145º04'E; WAM T74441 • 1 female, 1 juv.; Horsham; 36º43'S, 142º12'E; MV K-9368 • 1 female; Kangaroo Ground; 37º41'S, 145º13'E; MV K-9384 • 1 female; Kennington; 36º46'S, 144º18'E; MV K-14863 (CVIC 1173) • 1 male; Kilsyth, Mountfield Road; 37º48'S, 145º18'E; WAM T74440 • 1 male, 1 female, 5 juv.; La Trobe University; Bendigo; 36º46'48”S, 144º18'00”E; MV K-14859 (CVIC 1167) • 1 female; Laharum; Hawkers Gully; 36º56'S, 142º19'E; SAM • 3 males; Lake Mountain; 37º30'S, 145º52'E; MV K-9837 • 1 female; Lilydale; 37º45'S, 145º21'E; SAM • 1 female; Little Desert; 36º33'S, 141º50'E; MV K-9372 • 1 male; Little River; Wulgulmerang; 37º04'S, 148º15'E; MV K-9841 • 3 males; Macedon; 37º25'S, 144º33'E; NHMUK 1924.III.1.372–375 • 1 female; Melbourne; 37º49'S, 144º58'E; MV K-9426 • 1 female; Mitta Mitta River; 36º30'S, 147º26'E; MV K-9456 • 1 female; Moe; 38º11'S, 146º15'E; MV K-9399 • 1 female; Mordialloc district; 37º59'S, 145º05'E; MV K-9470 • 2 females; Mornington; 38º13'S, 145º02'E; MV K-9370); 1 female; same collection data as preceding; MV K-9480 • 1 female; Morwell National Park; 38º21'50''S, 146º24'58''E; SAM NN24287 • 1 female; Morwell National Park; 38º22'S, 146º25'E; SAM • 1 female; Mt Ben Cairn and Mt Donna Buang; 37º42'S, 145º39'E; QM • 3 females; Mt Buffalo; 36º42'S, 146º47'E; AM KS33319 • 1 male; Mt Buffalo; 36º46'S, 146º46'E; MV K-9849 • 1 female; Mt Buffalo, summit; 36º46'S, 146º46'E; MV K-9400 • 2 females; Mt Donna Buang; 37º42'S, 145º41'E; AM KS.33357; MV K9385 • 1 female; Mt Hotham; 36º58'S, 147º08'E; MV K-9398 • 1 female; Mt Pinnibar, summit; 36º31'S, 147º57'E; MV K-9715 • 1 female; Mulgrave; 37º55'S, 145º10'E; MV K-9474 • 1 female; Narracan; 38º15'S, 146º13'E; MV K-9378 • 3 females, 6 juv.; same collection data as preceding; MV K-9447 • 1 female; Newmerella; 37º44'S, 148º26'E; SAM • 1 female; Nunawading; 37º49'S, 145º10'E; MV K-9373 • 1 male, 2 females; Oakleigh; 40 Darling St.; 37º54'S, 145º06'E; WAM T73540 • 1 female; Ringwood; 37º48'S, 145º13'E; MV K-9446 • 1 male, 3 juv.; same collection data as preceding; MV K-9853 • 1 female, 2 juv.; same collection data as preceding; MV K-9395 • 1 male; Ringwood East, bushland cr Hume St and Walhalla Drive; 37º49'11''S, 145º15'41''E; WAM T100137 • 1 female; Sale; 38º06'S, 147º04'E; MV K-9371 • 14 females, Snobs Creek; 37º15'S, 145º52'E; MV K-9436 • 2 females; Spring Gully; 36º48'S, 144º17'E; MV K-14857 (CVIC 1176) • 2 females; Stoney Creek; South Gippsland; 38º36'S, 146º01'E; MV K-9411, K-9776); 1 female, Strathbogie; 36º51'S, 145º44'E; MV K-9380 • 1 female; Sunday Island; 38º42'S, 146º37'E; MV K-9412 • 1 female; Swan Hill; 35º20'S, 143º33'E; MV K-9419 • 1 male; Tarwin Lower; 38º42'S, 145º52'E; MV K-9836 • 1 male, 2 females, Wanganderry; 36º18'S, 146º14'E; MV K-14865 (CVIC 1155) • 1 male; same collection data as preceding; MV K-14862 (CVIC 1157) • 1 male; same collection data as preceding; MV K-14860 (CVIC 1159) • 1 male; same collection data as preceding; MV K-14864 (CVIC 1160) • 1 male; Warburton; 37º45'S, 145º42'E; MV K-9835 • 1 male, 2 females; same collection data as preceding; MV K-9458 • 1 female; Whipstick, Skylark Road; 36º37'30''S, 144º16'00''E; MV K-14866 (CVIC 1171) • 1 female; Whisky Flat; 36º19'S, 146º49'E; MV K-9461 • 1 male; Wirrate; near Mt Black Flora Reserve; 36º47'S, 145º00'E; MV K-14867 (CVIC 1163) • 1 female, 1 juv.; same collection data as preceding; MV K-14861 (CVIC 1164) • 3 females; Wodonga; 36º07'S, 146º53'E; MV K-9409 • 4 males; Woodend; 37º21'S, 144º31'E; MV K-9718 • 2 males, 2 juv.; same collection data as preceding; MV K-9401 • 3 males, 1 juv.; Yarra Glen; 37º39'S, 145º22'E; QM. **NEW ZEALAND** – • 2 females; New Zealand; no exact locality; NHMUK 1890.7.1.4183–84; **North Island** • 2 females; Bay of Plenty; Mokoia Island; Lake Rotorua; 38°04'48"S, 176°15'36"E; MONZ AS.004774 • 1 female; Greerton; 37°43'54"S, 176°08'31"E; MPI T17_0093 • 1 female; Henderson; 36°53'08"S, 174°38'13"E; MPI T17_00436 • 1 male; Lindemanns Loop Track; 37°32’S, 175°52’E; CMNZ 2020.67.38 • 1 female; Papamoa Beach; 37°43'12"S 176°20'49"E; MPI T16_00653 • 1 female; Raukawa St; Stokes Valley; Lower Hutt; 41°11’11”S, 174°58’45”E; MONZ AS.004798 • 1 female; Turangi; 38°58'20"S 175°48'51"E; MPI T18_00095; **South Island** • 1 female; Islington; 43°32’27”S, 172°30’47”E; CMNZ 2017.24.1 • 1 male, 3 females; Lincoln University; 43°38’53”S, 172°27’50”E (LUNZ).

***Salsa brisbanae* comb. nov.**

AUSTRALIA – **New South Wales** • 38 females, 17 juv.; ‘Allambi’ Telegraph Point; 31º19'S, 149º15'E; AM KS.56961 • 2 females; 1 juv.; same collection data as preceding; AM KS.56964 • 4 females, 1 juv.; same collection data as preceding; AM KS.56965 • 9 females, 3 juv.; same collection data as preceding; AM KS.56966 • 1 male; Antonio; 33º32'S, 150º01'E; AM KS.45148 • 2 females; Avalon; 33º38'S, 151º20'E; AM KS.32939 • 1 female; Barren Grounds Fauna Reserve; 34º40'S, 150º42'E; AM KS.65028 • 1 male; Beecroft; 33º45'S, 151º04'E; AM KS.45757 • 1 female; Beecroft, Observatory Park; 33º45'S, 151º04'E; AM KS.71741 • 1 female; Bellevue Hill, Sydney; 33º53'S, 151º17'E; AM KS.70046 • 1 male, 1 female; Bellingen River Cabins; ca. 5 km ESE of Bellingen; 30º29'S, 152º56'E; AM KS.65793 • 2 females, 1 juv.; Berry; 34º47'S, 150º42'E; AM KS.34172 • 1 female; Bonney Hills; 1 km S; 31º36'S, 152º51'E; AM KS.10165 • 1 female; Booti Booti National Park; 32º14'28''S, 151º32'50''E; AM KS.65319 • 1 female; Botany; 33º57'S, 151º12'E; AM KS.34057 • 2 females; Brooklana; E Dorrigo; 30º16'S, 152º51'E; AM KS.32675 • 2 females, 3 juv.; same collection data as preceding; AM KS.32688 • 1 male; same collection data as preceding; AM KS.34055 • 3 females, 1 juv.; same collection data as preceding; AM KS.34094 • 1 female; same collection data as preceding; AM KS.34099 • 1 female; Colo Vale; 34º24'S, 150º29'E; AM KS.32882 • 1 female; Cundletown; 31º54'S, 152º33'E; AM KS.56726 • 1 male; same collection data as preceding; AM KS.56727 • 1 female; same collection data as preceding; AM KS.56728 • 1 female; same collection data as preceding; AM KS.56729 • 1 female; same collection data as preceding; AM KS.56730 • 1 male; Elands; 31º38'S, 152º18'E; AM KS.45486 • 1 male; same collection data as preceding; AM KS.45730 • 1 female; same collection data as preceding; AM KS.46004 • 1 female; same collection data as preceding; AM KS.46005 • 1 male; Jamberoo Mountain; 34º39'S, 150º46'E; AM KS.54104 • 1 female; same collection data as preceding; AM KS.50221 • 1 male; same collection data as preceding; AM KS.56905 • 1 female; Kempsey ; banks of Mackay River; 31º05'S, 152º50'E; AM KS.56992 • 1 male, 1 female; Kiama; 34º40'S, 150º51'E; AM KS.32925 • 1 male, 1 female; same collection data as preceding; AM KS.32926 • 1 female; Lithgow; 33º29'S, 150º08'E; AM KS.32689 • 1 male; Minnamurra Falls; 34º38'S, 150º44'E; AM KS.34155 • 1 female; Mt Pleasant; near Wollongong; 34º26'S, 150º53'E; AM KS.32682 • 1 male; Munmorah National Park; 33º13'S, 151º34'E; AM KS.81977 • 1 female; Munmorah State Reserve; 33º12'26''S, 151º34'37''E; AM KS.65318 • 1 male; Myall Lakes National Park; 32º34'45''S, 152º17'27''E; AM KS.65317 • 1 male; same collection data as preceding; AM KS.65320 • 1 female; same collection data as preceding; AM KS.65321 • 1 female; Myuna Bay; Lake Macquarie; 33º03'S, 151º33'E; AM KS.68572 • 2 females; Narrabeen Lakes; 33º43'S, 151º17'E; AM KS.33299 • 1 female; Pacific Palms; 32º21'S, 152º31'E; AM KS.54225 • 1 male; Palm Beach; 30 km N Sydney; 33º35'10''S, 151º19'32''E; collno35 12232 • 1 male, 1 juv.; Petersham; 33º53'S, 151º9'E; AM • 1 male; Pittwater; 33º38'S, 151º18'E; AM KS.34121 • 2 females, 1 juv.; Richmond River; 28º55'S, 152º58'E; AM KS.32687 • 1 male; same collection data as preceding; AM KS.131086 • 1 female; same collection data as preceding; AM KS.131087 • 1 male; Royal National Park; 34º9'20''S, 150º03'30''E; AM KS.69038 • 1 female; Scalloway; Gerringong; 34º44'S, 150º47'E; SAM • 1 female; South West Rocks; 30º33'S, 153º02'E; AM KS.56013 • 1 female; same collection data as preceding; AM KS.56753 • 1 female; same collection data as preceding; AM KS.56756 • 1 female; same collection data as preceding; AM KS.56757 • 1 female; same collection data as preceding; AM KS.56758 • 1 female; same collection data as preceding; AM KS.56759 • 1 female; same collection data as preceding; AM KS.56760 • 1 female; same collection data as preceding; AM KS.56761 • 1 male; same collection data as preceding; AM KS.56762 • 1 male; St Georges Basin; 35º05'S, 150º35'E; AM KS.22647 • 3 males, 4 females; Sydney; 33º53'S, 151º13'E; NMV 1890.7.1.4173–9 • 1 female; Sydney; 33º53'S, 151º13'E; ZMH • 1 male; Sydney Botanical Gardens; 33º53'S, 151º13'E; AM KS.43038 • 1 female; Taree; 18km N; 31º48'S, 152º29'E; AM KS.10185 • 1 male; same collection data as preceding; AM KS.10187 • 1 female; Terrigal, Wamberal Lagoon; 33º25'S, 151º27'E; AM KS.32165 • 1 female, 1 juv., Tubrabucca; 31º58'S, 151º28'E; AM • 1 female; West Head; Ku-Ring-Gai; 33º35'S, 151º18'E; AM KS.33509 • 1 female; Wilton Park; QM • 1 female; Wyangarie; 29º24'S, 150º33'E; AM KS.32676 • 1 female; Yarramundi; 33º37'S, 150º40'E; AM KS.34162; **Queensland** • 1 male; Atherton Plateau; Rose Gums Wilderness Retreat; 12.4 km 059 ENE of Malanda; 17º18'51''S, 145º42'9''E; NHMD 12231 • 1 female; Atherton Tablelands; Rose Gums; 17º18'44''S, 145º42'9''E; NHMD 12233 • 1 female; Bakers Blue Mountain; 17km W Mt Molloy; 16º42'S, 145º10'E; QM • 1 female, 1 juv.; Bellenden Ker Range; 17º16'S, 145º51'E; QM S27961 • 1 female; Boonjee; 17º24'S, 145º44'E; QM • 1 female; Brighton; 27º19'S, 153º04'E; QM S26027 • 1 female; same collection data as preceding; QM • 1 male; Brisbane Forest Park; 27º25'04''S, 152º49'48''E; AM KS.69552 • 1 female; Burleigh Heads; 28º05'S, 153º27'E; MV K-10364 • 1 female; Burnett Creek; Boonah; 28º00'S, 152º41'E; QM • 1 female; Camira; 27º38'S, 152º55'E; QM • 1 female; same collection data as preceding; QM • 1 male, 2 females; Clarke Range; Cathu State Forest; 20º52'S, 148º36'E; QM • 1 female; Coen; 56 km NE of Attack Creek track crossing; 13º34'S, 143º27'E; AM KS.3470 • 1 male; Coloundra; 26º48'S, 153º08'E; AM KS.34200 • 1 male; Coolum; 26º34'S, 153º05'E; MV K-9857 • 1 female; Dalrymple Heights; near Eungella; 21º04'S, 148º35'E; AM KS.69317 • 1 male; same collection data as preceding; AM KS.69320 • 1 female; Darra; QM • 1 male; Dicky Beach; Caloundra; 26º48'S, 153º08'E; QM • 1 male; Echo Point Lookout; Lamington National Park; 28º16'S, 153º10'E; QM • 1 male, 2 females, 1 juv.; Eumundi; 26º29'S, 152º57'E; QM • 1 female; same collection data as preceding; QM • 1 female; Eungella area; Snake Road; 6.5km NW of Dalrymple Road; 21º02'15''S, 148º31'57''E; AM KS.58703 • 1 female, 1 juv.; Gordonvale; 17º06'S, 145º47'E; AM KS.32878 • 1 female; Holland Park; 27º28'S, 153º01'E; QM S30150 • 1 female; Kroombit Tops (Lower Dry Creek); 24º22'S, 150º57'E; QM • 1 female; Kroombit Tops (Upper TA47 Creek); 45km SSW Calliope; 24º22'S, 150º57'E; QM • 1 male, 1 female; same collection data as preceding; QM • 2 males; Lamington National Park; 28º12'S, 153º10'E; QM • 1 male, 3 females; Malanda; SW; Lot 2 Merragallan Road; 17º25'08''S, 145º32'37''E; AM KS.86087 • 2 males, 1 female; same collection data as preceding; AM KS.86105 • 13 females; Maleny; 26º46'S, 152º51'E; QM • 1 male; Manly; Brisbane; 27º28'S, 153º11'E; QM • 1 female; Massey Range; 6 km NW of Centre Bellenden Ker; 17º14'S, 145º48'E; QM S58618 • 1 female; Mt Aberdeen; N summit; 20º12'S, 147º55'E; QM S38612 • 1 female; Mt Bartle-Frere; 17º23'S, 145º49'E; QM • 1 male; Mt Elliot National Park, Upper North Creek, 19º29'S, 146º58'E; QM • 1 female; Mt Finnigan Summit; via Helenvale; 15º49'S, 145º17'E; QM • 1 female; Mt Gravatt; 27º33'S, 153º04'E; QM • 1 female; Mt Huntley, 28º08'S, 152º26'E; QM S49378 • 1 female; Mt Superbus; 28º14'S, 152º29'E; QM S15989 • 2 females; Mt Tamborine; 27º56'S, 153º12'E; QM • 1 female; same collection data as preceding; QM • 1 female; same collection data as preceding; 3º12'E; QM • 1 female; Mt William lower slopes; Dalrymple Heights; near Eungella; 21º01'S, 148º36'E; AM KS.12875 • 1 female, 1 juv.; same collection data as preceding; AM KS12877 • 1 female; Mt Williams summit; 16º55'S, 145º40'E; QM S41812 • 1 male; Mulgowie; 27º44'S, 152º22'E; QM • 1 male; North Tamborine; 27º54'S, 153º11'E; QM • 1 female; same collection data as preceding; QM • 2 males; Peak Downs; 22º56'S, 148º05'E; ZMH • 2 females; Peak Downs; 22º56'S, 148º05'E; NHMUK 1890.7.1.4173–4 • 1 male, 2 females, 2 juv.; Ravensbourne; 27º22'S, 152º11'E; QM • 1 male; Ravensbourne National Park; 27º22'S, 152º11'E; QM • 1 female; Ravenshoe; 17º37'S, 145º29'E; QM • 1 male, 1 juv.; Ravenshoe; 17º37'S, 145º29'E; QM • 1 female; Ternvale; near Gatton, 27º34'S, 152º20'E; QM) **Tasmania:** 1 male, New Town; 42º53'S, 147º19'E; AM); 5 females, 2 juv.; Orford; 42º34'S, 147º52'E; AM; **Victoria** • 1 male; no exact locality; MV K-9858 • 1 male, 13 females; Berwick; Quarry Hill; 38º02'S, 145º20'E; QM • 1 female; Chiltern; 36º9'S, 146º36'E; MV K-9860 • 1 female; Hazelwood South; 38º20'S, 146º27'E; SAM • 1 male; Mornington; 38º13'S, 145º02'E; MV K-9345 • 1 male, 9 females; Mornington; 38º13'S, 145º02'E; MV K-9866.

***Salsa canalae* comb. nov.**

NEW CALEDONIA – • 1 male; Mt Koghis Hotel; 22º11'S, 166º32'E; WAM T75922 • 1 female; Mt Koghis Hotel; 22º11'S, 166º32'E; WAM T75921 • 1 female; Mt Ouin; 22º01'S, 166º27'E; WAM T75923 • 1 female; Mt Panié; 20º33'S, 164º47'E; AM KS.54210.

***Salsa recherchensis* comb. nov.**

AUSTRALIA – **South Australia** • 1 female; Bald Hill Beach; near Port Wakefield; 34º15'S, 138º10'E; SAM • 1 male; Coffin Bay; behind caravan park; 34º36'S, 135º28'E; SAM • 2 males, 8 females; Dudley Conservation Park; Kangaroo Island; 35º48'S, 137º51'E; SAM • 2 males, 2 females, 2 juv.; Kirton Point; Port Lincoln; 34º43'S, 135º52'E; SAM • 1 female; Nappyalla; 35º20'S, 139º07'E; SAM • 1 male; Penneshaw; Kangaroo Island; 35º48'S, 137º52'E; SAM • 1 female, 1 juv.; between Pennington Bay and Point Reynold; Kangaroo Island; 35º52'S, 137º44'E; SAM NN24286 • 1 male, 1 female; Port Germein; 33º01'S, 138º00'E; SAM • 1 female; Port Germein; caravan park; 33º01'S, 138º00'E; SAM • 7 males, 13 females, 3 juv.; Tod River mouth; N of Port Lincoln; 34º36'S, 135º54'E; SAM • 2 females, 3 juv.; West Island; SW Victor Harbour; 35º36'S, 138º35'E; SAM; **Western Australia** • 1 male; Albany; 35º02'S, 117º53'E; WAM T73581 • 1 female; Bald Island; 34º55'S, 118º27'E; WAM T101323 • 1 female; Booanya; 32º45'S, 123º36'E; MV K-9831 • 1 female; Bremer Bay; near Mt Henry; 34º28'42''S, 119º22'21''E; WAM T77351 • 1 female; same collection data as preceding; WAM T77362 • 1 male, 1 female, 2 juv.; Bunbury; Busselton Road (no exact locality); SAM • 2 males; Busselton; 33º39'S, 115º20'E; AM KS.32872 • 2 females, 2 juv.; same collection data as preceding; AM KS. 32685) • 1 male; same collection data as preceding; AM KS.34330 • 1 male; Capel; lot 7 NW Road; 33º33'S, 115º33'E; WAM T73696 • 4 females, 1 juv.; Cheynes Beach; 34º53'S, 118º25'E; WAM T73560 • 1 female; Glenbourne; 33º54'S, 115º00'E; WAM T75928 • 1 male, 1 female; Jorndee Creek Campsite; Cape Arid National Park; 33º54'44''S, 123º20'08''E; WAM T81445 • 1 female; same collection data as preceding; WAM T81446 • 1 female; Margret River area; Burnside; 2–3 km North of Walcliffe Road; 33º56'S, 115º01'E; AM KS.15106 • 1 male; Orleans Bay caravan park; 33º56'S, 122º36'E; WAM T73532 • 5 females; Perth; 31º57'S, 115º51'E; AM KS.34112 • 1 male, 1 female; Pyungoorup; 34º22'S, 118º19'E; WAM T81466 • 1 female; Quaalup Homestead; 34º17'S, 119º26'E; WAM T85258 • 2 females; Sabina River; 33º39'S, 115º24'E; WAM T77199 • 1 female, 3 juv.; same collection data as preceding; WAM T77244 • 9 females; same collection data as preceding; WAM T77253 • 1 female, 1 juv.; same collection data as preceding; WAM T77259 • 2 females; same collection data as preceding; WAM T77263 • 1 female; same collection data as preceding; WAM T77271 • 4 females, 1 juv.; same collection data as preceding; WAM T77294 • 1 female; same collection data as preceding; WAM T77295 • 13 females; same collection data as preceding; WAM T77296 • 1 male, 3 females, 2 juv.; same collection data as preceding; WAM T77297 • 1 female, 1 juv.; same collection data as preceding; WAM T77298 • 8 females; same collection data as preceding; WAM T77299 • 6 females, 8 juv.; same collection data as preceding; WAM T77300 • 2 females, 12 juv.; same collection data as preceding; WAM T77301 • 2 females; same collection data as preceding; WAM T77302 • 4 females, 4 juv.; same collection data as preceding; WAM T77303 • 2 females, 1 juv.; same collection data as preceding; WAM T77304 • 4 females; same collection data as preceding; WAM T77305 • 1 female; same collection data as preceding; WAM T77306 • 20 females, 83 juv.; same collection data as preceding; WAM T77307 • 42 females; same collection data as preceding; WAM T77308 • 46 females; same collection data as preceding; WAM T77309 • 19 females; same collection data as preceding; WAM T77310 • 28 females, 31 juv.; same collection data as preceding; WAM T77311 • 2 males; same collection data as preceding; WAM T77312 • 13 females, 11 juv.; same collection data as preceding; WAM T77313 • 14 females, 3 juv.; same collection data as preceding; WAM T77314 • 1 female; same collection data as preceding; WAM T77329 • 1 female; same collection data as preceding; WAM T77330 • 1 female; Salisbury Island; Recherche Archipelago; 34º22'S, 123º33'E; WAM 92/2120 • 1 female; same collection data as preceding; WAM 92/2122 • 1 female; same collection data as preceding; WAM T75224 • 1 female; Shannon River at Nelson; 34º43'S, 116º21'E; WAM T74435 • 1 female; same collection data as preceding; WAM T75229 • 2 females; Stirling Range National Park; Bluff Knoll; 34º22'51''S, 118º15'02''E; WAM T99104 • 3 males; same collection data as preceding; WAM T99854 • 1 female; Stirling Range National Park; Ellen Peak Trail; 34º23'S, 118º17'E; WAM T81440 • 1 female; Torndirrup National Park; gully outside N edge; on Limeburners Road; 35º05'S, 117º54'E; WAM T73546 • 1 female; Torndirrup National Park; Quaranup Road; 35º05'S, 117º55'E; WAM T74443 • 1 male; Valley of the Giants; 34º58'S, 116º53'E; SAM • 1 female; Waroona; 32º50'S, 115º55'E; MV K-9428 • 1 female; Wichcliffe; Golgotha Cave; 34º06'S, 115º02'E; AM KS.34329.

***Salsa rueda* sp. nov.**

AUSTRALIA – **Australian Capital Territory** • 1 female; Canberra; 35º18'S, 149º08'E; QM; **New South Wales** • 1 female; ‘Tuglo’; near Singleton; Carrow Brook; 32º14'S, 151º16'E; AM KS.8501 • 1 female; Jamberoo; 34º39'S, 150º47'E; AM KS.50201 • 1 female; Jamberoo Mountain; 34º40'S, 150º43'E; AM KS.65666 • 1 female; same collection data as preceding; AM KS.56742 • 1 female; same collection data as preceding; AM KS.70916 • 1 male; Jenolan Caves; 33º49'18''S, 150º01'20''E; AM KS.32838 • 1 male, 1 female, 1 juv.; Lithgow; 33º29'S, 150º08'E; AM KS.34144 • 1 male; Tinderry Range; 17.8 km from Captains Flat Rd; on Tinderry Road; 35º45'01''S, 149º15'40''E; WAM T67901 • 1 male, 2 females; Tomalla; 31º48'S, 151º28'E; MV K-9770 • 1 male, 1 female; Tubrabucca; 31º52'S, 151º25'E; MV K-9477 • 1 female; same collection data as preceding; MV K-9722 • 2 females; same collection data as preceding; MV K-9792 • 1 male; same collection data as preceding; MV K-14856; **Tasmania** • 1 female; George Town; 41º06'S, 146º'E; SAM; **Victoria** • 1 male; Port Albert; 38º41'S, 146º42'E; MV K-9851.

***Salsa tartara* sp. nov.**

AUSTRALIA – **New South Wales** • 1 female; Lord Howe Island; 31º34'42''S, 159º04'30''E; AM KS.34007 • 1 male; Lord Howe Island; no exact locality; AM KS.98751 • 1 female; Lord Howe Island; coastal track from Cobby`s Corner; 31º32'43''S, 159º04'54''E; AM KS.88731 • 1 female; Lord Howe Island; foot of saddle rise; Erskine Valley; 31º34'42''S, 159º04'30''E; AM KS.34147 • 1 female; Lord Howe Island; track to start of Mt Gower Track; S end Salmon Beach; 31º33'50''S, 159º04'30''E; AM KS.70617 • 1 female; Lord Howe Island; track to Valley of the Shadows; 31º31'50''S, 159º04'32''E; AM KS.70661.
